# Glucocorticoids promote transition of ductal carcinoma in situ to invasive ductal carcinoma by inducing myoepithelial cell apoptosis

**DOI:** 10.1186/s13058-018-0977-z

**Published:** 2018-07-04

**Authors:** Arantzazu Zubeldia-Plazaola, Leire Recalde-Percaz, Núria Moragas, Mireia Alcaraz, Xieng Chen, Mario Mancino, Patricia Fernández-Nogueira, Miquel Prats de Puig, Flavia Guzman, Aleix Noguera-Castells, Anna López-Plana, Estel Enreig, Neus Carbó, Vanessa Almendro, Pedro Gascón, Paloma Bragado, Gemma Fuster

**Affiliations:** 1 Institut d’Investigacions Biomèdiques August Pi i Sunyer (IDIBAPS), Barcelona, Spain; 2 Department of Medicine, University of Barcelona, Barcelona, Spain; 3 Department of Medical Oncology, Hospital Clínic, Barcelona, Spain; 4 Department of Senology, Clínica Planas, Barcelona, Spain; 5 Histopathology-Citology, Anatomical Pathology Service, Centro Médico Teknon, Barcelona, Spain; 6 Department of Biochemistry and molecular Biomedicine, University of Barcelona, Barcelona, Spain; 7 Division of Medical Oncology, Department of Medicine, Harvard Medical School, Dana-Farber Cancer Institute, Brigham and Women’s Hospital, Boston, MA USA

**Keywords:** Glucocorticoids, DCIS, Invasiveness, Myoepithelial cells, Apoptosis

## Abstract

**Background:**

The microenvironment and stress factors like glucocorticoids have a strong influence on breast cancer progression but their role in the first stages of breast cancer and, particularly, in myoepithelial cell regulation remains unclear. Consequently, we investigated the role of glucocorticoids in ductal carcinoma in situ (DCIS) in breast cancer, focusing specially on myoepithelial cells.

**Methods:**

To clarify the role of glucocorticoids at breast cancer onset, we evaluated the effects of cortisol and corticosterone on epithelial and myoepithelial cells using 2D and 3D in vitro and in vivo approaches and human samples.

**Results:**

Glucocorticoids induce a reduction in laminin levels and favour the disruption of the basement membrane by promotion of myoepithelial cell apoptosis in vitro. In an in vivo stress murine model, increased corticosterone levels fostered the transition from DCIS to invasive ductal carcinoma (IDC) via myoepithelial cell apoptosis and disappearance of the basement membrane. RU486 is able to partially block the effects of cortisol in vitro and in vivo. We found that myoepithelial cell apoptosis is more frequent in patients with DCIS+IDC than in patients with DCIS.

**Conclusions:**

Our findings show that physiological stress, through increased glucocorticoid blood levels, promotes the transition from DCIS to IDC, particularly by inducing myoepithelial cell apoptosis. Since this would be a prerequisite for invasive features in patients with DCIS breast cancer, its clinical management could help to prevent breast cancer progression to IDC.

**Electronic supplementary material:**

The online version of this article (10.1186/s13058-018-0977-z) contains supplementary material, which is available to authorized users.

## Background

Breast cancer, the most frequent tumour among women worldwide, is a heterogeneous disease [1]. The most common non-invasive breast cancer lesion is ductal carcinoma in situ (DCIS) [2], defined as intraductal, since there is clonal proliferation of cancerous epithelial cells within the ductal lumen without spreading into the mammary stroma and with the myoepithelial cell layer and basement membrane (BM) remaining intact [2]. However, DCIS is considered a non-obligatory precursor lesion of invasive ductal carcinoma (IDC), in which the disappearance of myoepithelial cells and the BM leads to the invasion of the mammary stroma by tumour epithelial cells [3]. DCIS is a highly heterogeneous lesion and the evolution differs in each patient: some rapidly progress to IDC if untreated or undertreated, whereas others remain virtually unaltered for 5–20 years or never progress [4]. Epidemiologically, DCIS accounts for 15–25% of newly diagnosed breast cancer cases in the USA and this incidence is increasing [5], while the frequency of IDC remains stable [6], indicating that some DCIS will be over treated without therapeutic benefit. Therefore, there is a need to improve the management of patients with DCIS by matching the risk to each individual, avoiding over/under treatment of patients with DCIS to prevent the transition to IDC, which is less therapeutically affordable than DCIS.

Recent efforts have focused on unravelling the role of cancerous epithelial cells in DCIS and IDC [4, 7]. However, the mechanisms regulating the transition from DCIS to invasive carcinoma remain largely unknown, hampering correct subsequent surgery and treatment. The characteristic feature in the transition from DCIS to IDC is the disappearance of the myoepithelial cell layer and its BM [8], which is closely related to the loss of function of myoepithelial cells, since the gene expression of the main component of the BM, laminin, is altered [9]. Myoepithelial cells play a crucial role as tumour suppressors of the transition to the invasiveness of DCIS [10]. However, despite recent progress in cell research [11–13], the molecular mechanisms that regulate myoepithelial cell layer disruption in the transition from DCIS to IDC remain unexamined.

Recent studies have highlighted the role of the microenvironment in the transition from DCIS to IDC [14–16]. During the transition from DCIS to IDC, epigenetic changes have been observed in stromal cells, including fibroblasts and myoepithelial cells [17], suggesting that the microenvironment plays a relevant role. The tumour microenvironment may be modulated by stress response factors such as hormones and neuronal factors [18]. In fact, the effect of stress-related neuroendocrine factors on breast cancer progression has been addressed by several research groups including ours [19–21]. However, the impact of these molecules in the transition from DCIS to IDC has not yet been studied. Thus, understanding how stress and associated neuronal factors affect the biology of DCIS and, in particular, the fate of myoepithelial cells, could be relevant to clinical management and outcomes.

Glucocorticoids are one of the most important neuronal factors involved in the stress response, and are implicated in other physiological functions, such as the inflammatory response and glucose metabolism regulation [22]. Glucocorticoids are lipophilic molecules and diffuse across the cell membrane. They act through their intracellular glucocorticoid receptors (GR) and mineralocorticoid receptors, although it is suggested they induce tumour progression activity in a GR-dependent manner [23].

Cortisol and corticosterone are the two major glucocorticoids present in humans. Blood corticosterone levels are 10–20-fold lower than cortisol, but corticosterone represents almost 40% of total active glucocorticoids in cerebrospinal fluid [24]. Corticosterone is the major glucocorticoid in rodents and has been related to cancer progression in animal models [25], although its role in the context of DCIS invasiveness is unknown.

Cortisol, the major glucocorticoid found in humans, generates a physical response to the stress signal by binding to its receptor, GR. Cortisol plays an important role in mammary gland development [26] and function and therefore increased levels caused by stress may affect the fate of the mammary tissue. In addition, cortisol has an impact on oestrogen activity in the mammary gland, inducing aromatase activity [27] and regulating breast cancer [19]. Cortisol is an immunomodulator that reduces the immune system’s ability to detect and respond to tumour cells [19, 28], acts on DNA repair mechanisms, and modulates apoptosis [19, 23, 29].

GR is expressed in healthy breast tissue and in breast cancer, including DCIS and IDC [30]. GR expression diminishes with breast cancer progression and thus is higher in DCIS than in IDC [30]. Moreover, the GR antagonist, RU486 or mifepristone, has been suggested to be a normal breast epithelium protector [31], and in this context, there is an ongoing clinical trial based on the activity of RU486 in patients with breast cancer genes 1/2 (*BRCA1*/*2*) (clinical trial identifier NCT01898312). Additionally, pathological studies have found that myoepithelial cells express higher levels of GR than epithelial cells [30]. However, studies of the function of cortisol in the fate of myoepithelial cells and in the regulation of DCIS invasiveness are lacking. The purpose of this study was to investigate the effects of glucocorticoids on the transition from DCIS to IDC, paying special attention to myoepithelial cell modulation. Interestingly, our studies demonstrate that glucocorticoids foster the transition from DCIS to IDC through the induction of myoepithelial cell apoptosis and the reduction in laminin levels in in vitro and in vivo stress model experiments. Furthermore, in patients with breast cancer, myoepithelial cell apoptosis is more frequent in patients with DCIS+IDC than in patients with DCIS, implying this process may be a key factor in the evolution from DCIS to IDC.

## Methods

### Cell culture

MCF10DCIS (Asterand, MI, USA) and MCF10A (American Type Culture Collection (ATCC), VA, USA) human mammary cell lines were cultured at 37° in a humidified atmosphere with 5% CO_2_ in Dulbecco’s modified Eagle’s medium nutrient mixture-F12 (DMEM-F12) (Gibco, Life Technologies, CA, USA), supplemented with 1% of L-GlutaMAX™ 200 mM (Gibco, Life Technologies CA, USA), 100 U/mL penicillin, 100 μg/mL streptomycin and 5 μg/mL Fungizone (PSF) (Gibco, Life Technologies CA, USA), 5% of horse serum (Gibco, Life Technologies CA, USA) and, specifically, for MCF10A, 20 ng/mL epithelial growth factor (EGF) (Peprotech, NJ, USA), 0.5 μg/mL hydrocortisone (Sigma, MO, USA), 10 μg/mL insulin (Sigma, MO, USA) and 100 ng/mL cholera toxin (Sigma, MO, USA) were also added.

Primary myoepithelial cells and epithelial cells were isolated from fresh healthy mammary tissue from five women aged 20–45 years (Additional file 1: Table S1). Samples were obtained under the approval of the Institutional Review Board of the Hospital Clinic and Clinica Planas, Barcelona. To obtain mammary myoepithelial cells, epithelial cells and organoid fractions were cultured in M87A medium [32] (see Additional file 2). Cell sorting of CD10+ cells from epithelial and organoid fractions was used to isolate myoepithelial cells, as described by LaBarge and colleagues [33].

### Cell viability

To analyse in vitro cell viability, 3-(4, 5-dimethylthiazol-2-yl)-2, 5-diphenyl tetrazolium bromide (MTT) assays (Promega, WI, USA) were performed. Briefly, 1.5*10^4^ cells/100 μL were seeded per well in MW96 plates. After 24 h, cells were treated for 48 h with increasing concentrations of cortisol (0.1, 0.25, 0.5, 1, 2.5, 5 and 10 μM), corticosterone (0.125, 0.25, 0.375, 0.5, 0.75, 1, 1.25 and 1.5 μM) or with the corresponding vehicle concentration (methanol). The analysis was carried out following the kit instructions (Promega, WI, USA). Absorbance was measured immediately using a microplate reader spectrophotometer (Sinergy, Bio Tek, VE, USA). Measurements were made at 492 nm (test wavelength) and at 620 nm (reference wavelength), to correct for noise.

### Cell cycle

Cell cycle was analysed using bromodeoxyuridine (5-Br-2′-deoxyuridine or BrdU): 2*10^5^ cells were seeded in p60 dishes and when 70% of cell density was achieved cells were treated with 0.7 μM of cortisol or vehicle (methanol). After 48 h the cells were treated with 10 μM BrdU for 5 h. Subsequently, BrdU (10 μM) was added to each well and left to be incorporated into the newly synthesized DNA of replicating cells for 3 h. Cells were harvested with PBS and fixed with ethanol O/N. The next day, cells were permeabilized and denaturalized by HCl 2 M and 0.5% Triton X-100 for 1 h at room temperature (RT); 0.1 M Na_2_B_4_O_7_ was then used to neutralize HCl for 20 min at RT. Anti-BrdU-fluorescein isothiocyanate (FITC) antibody (1:20, #F7210, Dako, Denmark) was added and the mixture kept in the dark for 1 h after PBS washing. Next, cells were suspended in 500 μL of propidium iodide (PI) buffer (PBS, 0.4 mg/mL RNase A, 0.2 μg/mL PI) and incubated in darkness for 15−20 min. The cells were analysed by flow cytometry (BD LSRFortessa) and the results evaluated by FACSDiva software (Becton-Dickinson, NJ, USA).

### Cell apoptosis

To determine cell apoptosis, an Annexin V assay (BD Annexin V: FITC Apoptosis Detection Kit I, (BD Biosciences, CA, USA) was performed in semi-confluent cell cultures: 3*10^5^ cells were seeded in p60 dishes until the plates were 70% confluent. The cells were then treated with 0.1, 0.25 and 1 μM cortisol or with 0.25, 0.6 and 1 μM of corticosterone for 48 h or vehicle (methanol). The assay was performed following the manufacturer’s instructions (BD Biosciences, CA, USA) and analysed using flow cytometry (FORTESSA LSR, Becton-Dickinson, NJ, USA). The results were evaluated by FACSDiva software (Becton-Dickinson).

### Three-dimensional cell culture

The functional characteristics of the cells and their ability to form proper acinar structures were evaluated by three-dimensional (3D) cultures using BD Matrigel™ Basement Membrane Matrix (BD Biosciences, CA, USA) under corticosterone and cortisol treatment. On-top 3D cell cultures were made in MW24 plates and 1*10^5^ of a mix of primary epithelial and myoepithelial cells/well were seeded in 500 μL of M87A medium supplemented with 4% of matrigel on a matrigel pre-coated MW24 well. Medium was changed three times per week and 1 mL of M87A medum with 4% of matrigel was added to each well. Cells were seeded on top of matrigel cultures and treatment was started after 5 days. Briefly, the cell medium was discarded and new M87A medium was mixed with the desired amount of the molecule of interest and 4% of matrigel on ice. Cortisol 0.7 μM, corticosterone 1 μM and RU486 0.5 μM (Sigma, MO, USA) was used as treatment. Treatment was administered three times per week until day 14 after seeding.

### In vivo experiments

In vivo experiments were performed in female NUDE mice aged 3–4 weeks that were bred at the medical school’s animal facility laboratory and kept under specific pathogen-free conditions at constant ambient temperature (22–24 °C) and humidity (30–50%). The mice had access to sterilized food and tap water *ad libitum*. In vivo experiments were performed according to Catalan Government Animal Experimentation Ethics Committee regulations (*Comitè Ètic d’Experimentació Animal*(CEEA)).

The MCF10DCIS cell line, which forms DCIS in mice and spontaneously evolves to invasive carcinoma, was used [18]. Mice were anaesthetized using ketamine/xylazine to inoculate the mammary fat pad with cancer cells: 10^5^ cells diluted in PBS-matrigel (BD Biosciences, CA, USA) (1:1) were then injected orthotopically into each mouse mammary fat pad with a total volume of 100 μL.

### In vivo time course

Tumour growth and animal weight were monitored twice a week for 29 days after MCF10DCIS inoculation. Mice were killed at days 7, 22 and 29 after inoculation. Tumours were extracted, fixed in paraformaldehyde (PFA) 4% and stored as paraffin-embedded tissue.

### In vivo immobilization stress model

Mice were subjected to immobilization stress 7 days after inoculation of MCF10DCIS cells into the mammary fat pad. Briefly, mice were placed into 50-mL Falcon tubes where various holes ensured proper ventilation for 2 h, 5 days per week, for 3 weeks [34, 35]. We consider our in vivo stress model a chronic stress model because acute stress is a type of punctual and short-term stress [36]. On the other hand, chronic stress implies a more repetitive and/or long-term exposure to the stress source [37]. In parallel, blood was extracted from the mouse tail vein twice per week, starting before the inoculation of cells and continuing until sacrifice. Blood was extracted using EDTA-coated eppendorf tubes (Microvette® CB 300, Sarstedt, Germany), kept on ice and then centrifuged at 10,000 rpm for 5 min at 4 °C. Plasma was recovered and stored at − 80 °C.

### Corticosterone ELISA in stress experiments in vivo

Circulating levels of corticosterone in murine plasma were measured using an ELISA kit according to the manufacturer’s instructions (Abnova, China). Colour intensity was measured at 450 nm in a spectrophotometer (Sinergy, BioTek, VE, USA). Corticosterone levels were determined using a standard sigmoidal curve and comparing it with the standard curve.

### In vivo chicken embryo model

The chicken chorioallantoic membrane (CAM) model has been previously described [38–40]. For the CAM xenografts we used premium specific pathogen-free (SPF), fertile, 9-day- incubated embryonated chicken eggs supplied by Gibert farmers: 2*10^6^ MCF10DCIS cells diluted in PBS and matrigel were inoculated on CAMs and tumours were grown for 6 days. The day after inoculation, the tumours were treated with cortisol 0.7 μM or/and RU486 0.5 μM for 5 days. On day 6 after inoculation the tumours were excised, weighed, measured and immediately fixed in 4% formaldehyde to perform IF analysis.

### Immunofluorescence

To carry out immunofluorescence in 2D cell culture systems, cells were seeded on coverslips, incubated at 37° for 24 h and treated for 24 h with 0.7 μM cortisol and 1 μM corticosterone. Immunofluorescence of K14 (1/100, #ab9220, Abcam, UK), K19 (1/10, #Troma-III, DSHB, IA, USA) and for GR distribution studies (1/100, #PA-1-511A, Thermo Fisher Scientific, MA, USA) was carried out as previously described [41]. After washing, secondary antibodies were added and cells were counterstained with Hoechst dye 2 μg/mL (Life Technologies, CA, USA) for 15 min at RT in a dark humidified chamber. Cell coverslips were mounted using ProLong® Gold antifade reagent (Life Technologies, CA, USA).

Three-dimensional immunofluorescence was carried out in 3D cell cultures for 14 days after 8 days treatment with cortisol 0.7 μM, corticosterone 1 μM and RU486 0.5 μM as previously described [41]. K14 (1/100), K19 (1/10) and laminin (1/100, #ab11575, Abcam, UK) were used as primary antibodies. Muc1-FITC (#559774, Becton Dickinson, NJ, USA) was used as primary antibody to counterstain epithelial cells. Incubation with secondary antibodies was sequentially applied after washing. Samples were then counterstained with Hoechst dye 2 μg/mL (Life Technologies, CA, USA) for 15 min and analysed by confocal microscopy (Leica TCS-SP5 Broadband Confocal and Multiphoton Microscope). Additionally, we performed immunofluorescence of α-smooth muscle actin (α-SMA) (1/100, #M0851, Dako, Denmark) in the tumour sections. Briefly, the slides were incubated at 65 °C for 30 min and hydrated following a decreasing ethanol gradient (100–70%). Then, citrate buffer (pH = 6.0) was used for immunoreactivity enhancement. The primary and secondary antibody incubation was performed as described above for the 2D immunofluorescence.

### Immunohistochemical analysis

Immunohistochemical analysis of tumour samples stored as paraffin-embedded tissue was carried as previously described [42]. Laminin (1/100) and cleaved caspase 3 (1/100, #9664S, Cell Signaling, MA, USA) antibodies were used. Depending on the technique performed and the manufacturer’s specifications, different antibodies were used to detect myoepithelial cells: CD10 (1/100, #M7308, Dako, CA, USA), p63 (1/100, #sc-8431, Santa Cruz Biotechnologies, TE, USA) and αSMA (1/100) were used as primary antibodies. After washing the samples were incubated in the presence of HRP-conjugated secondary antibodies. After washing, samples were incubated with Vectastain ABC for 30 min in a humidified chamber followed by incubation in the presence of DAB substrate (FAST™ 3,3′-diaminobenzidine tablets, Sigma) for 5–10 min at RT, monitoring colour development by microscopy. Slides were washed with water and counterstained in 1:3 Gill II haematoxylin (Panreac) for 1 min. The slides were mounted with Cytoseal™ 60 (Thermo Scientific), left to dry and analysed by phase contrast microscopy.

### Double immunofluorescence in paraffin-embedded tissue

The first steps for double immunofluorescence in paraffin-embedded tissue were identical to those of the immunohistochemical analysis. Deparaffinization and rehydration were carried out followed by antigen retrieval, as previously described [42]. After cooling, samples were washed with PBS and blocked with 10% normal goat serum in PBS for 10 min at RT. The primary antibodies, p63 (1/100) and cleaved caspase 3 (1/100) or laminin (1/100) were diluted in 5% normal goat serum in PBS and the mix was incubated for 2 h at RT in a humid box. After washing, the mix was incubated with secondary antibodies for 1 h at RT in a dark humid box. Slides were counterstained with Hoechst dye 2 μg/mL (Life Technologies, CA, USA) for 15 min in a dark humid box. Finally, samples were mounted with ProLong® Gold antifade reagent (Life Technologies, CA, USA) and analysed using fluorescence microscopy.

### Patient samples

Samples from patients with DCIS or DCIS+IDC were obtained from the Biobank of the Hospital Clinic of Barcelona, Institut d’Investigacions Biomèdiques August Pi i Sunyer (IDIBAPS), after Institutional Ethics Committee approval. Thirteen samples from patients with DCIS and fifteen from patients with DCIS+IDC were used (lesions specified in Additional file 3: Table S2). Myoepithelial cell layer apoptosis was evaluated using immunohistochemical analysis of CD10 (1/100) and double immunofluorescence of cleaved caspase 3 (1/300) and p63 (1/100).

### Statistical analysis

The results were plotted and analysed using GraphPad Prism7 software (CA, USA). The Mann-Whitney test, Wilcoxon paired test and one-way analysis of variance (ANOVA) followed by a post-hoc test were used according to the type of analysis.

## Results

### Cortisol inhibits myoepithelial cell growth through induction of cell cycle arrest and apoptosis

To study the effect of glucocorticoids on myoepithelial cells, we used human myoepithelial primary cell cultures isolated from reduction mammoplasties [41]. Since primary epithelial cells are difficult to maintain in culture, we used the MCF10A cell line as representative of the epithelial population. Another epithelial cell line, MCF10DCIS, which spontaneously generates DCIS when transplanted into mice [18], was also used. First, we tested the expression of glucocorticoid receptors in epithelial, myoepithelial and MCF10DCIS cells (Additional file 4: Figure S1). We found that all cell lines express the glucocorticoid receptor (GR), and that myoepithelial cells express higher levels than MCF10A or MCF10DCIS.

**Fig. 1:**
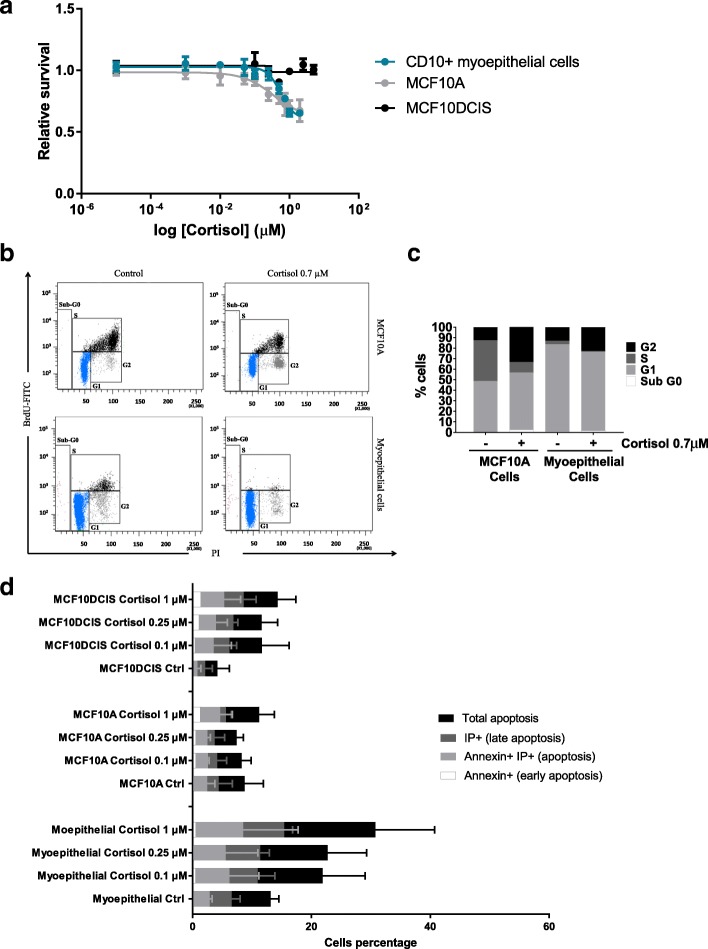
Cortisol effects on myoepithelial, MCF10A and MCF10DCIS cell viability, cell cycle and apoptosis. **a** Cell viability evaluation by 3-(4, 5-dimethylthiazol-2-yl)-2, 5-diphenyl tetrazolium bromide (MTT) assay after 48 h of increasing doses of cortisol treatment (0.1–10 μM). **b** Cell cycle distribution was determined by bromodeoxyuridine (BrdU)-fluorescein isothiocyanate (FITC) and Propidium iodide (PI) assay after 48 h of treatment with cortisol 0.7 μM or vehicle (methanol) and evaluated by flow cytometry. **c** Graphic representation of cell cycle distribution in percentages by the population evaluated. **d** Cell apoptosis in its different stages (early apoptosis, apoptosis and late apoptosis) was determined after cortisol treatment with 0–1 μM doses by the Annexin V method and measured by flow cytometry. All experiments were carried out in triplicate. The Mann-Whitney test was used for statistical analysis

Two-dimensional myoepithelial cells and epithelial cell cultures were then treated with different concentrations of cortisol for 48 h. MTT assays showed that myoepithelial cell survival was reduced dose-dependently in the presence of cortisol, reaching a reduction of almost 50% at the highest tested dose (Fig. 1a). Epithelial MCF10A cell growth was also slightly more attenuated after treatment with cortisol (Fig. 1a), suggesting it affects the proliferation of both epithelial and myoepithelial cell growth. MCF10DCIS epithelial cells were resistant to cortisol (Fig. 1a) at the maximum doses tested.

Since cortisol inhibits myoepithelial cell growth we used a BrdU assay to analyse its effect on the cell cycle. We found that treatment with cortisol arrested myoepithelial cells in both the G1 and G2 phases, further decreasing the number of cells undergoing the S phase (Fig. 1b-c). In contrast, in MCF10A cells, only a very small percentage of cells were arrested in the G2 phase and there was no arrest in the G1 phase. Therefore, the reduction in cells in the S phase was not as prominent as in the case of myoepithelial cells (Fig. 1b-c). These results suggest that myoepithelial cells are more susceptible than epithelial cells to the effects of cortisol on their cell cycle. Thus, cortisol inhibits epithelial and, in a stronger way, myoepithelial cell growth partially through the induction of cell cycle arrest.

However, the effect of cortisol on the cell cycle did not explain the differences in survival between myoepithelial and epithelial cells after glucocorticoid treatment. Therefore, we also investigated the induction of apoptosis by glucocorticoids. We used an Annexin V binding assay to determine whether these cells were undergoing apoptosis and found that myoepithelial cell apoptosis increased in a dose-dependent manner by around 15% (Fig. 1d): however, the percentage of apoptotic cells was always below 5% for MCF10A epithelial cells and the MCF10DCIS cell line (Fig. 1d). Thus, we speculate that the induction of apoptosis might explain the reduction observed in the survival of myoepithelial cells after glucocorticoid treatment, an effect that seems to be restricted to the myoepithelial cell lineage.

### Cortisol hampers the formation of 3D acinar-like structures in vitro

After observing that glucocorticoids inhibited the proliferation of myoepithelial cells by causing apoptosis and cell cycle arrest, we studied how glucocorticoids influenced the formation and structure of 3D *acini* using on-top matrigel cultures of a mix of healthy primary mammary epithelial cells and myoepithelial cells that, when grown in 3D systems, can form acinar structures [41].

**Fig. 2:**
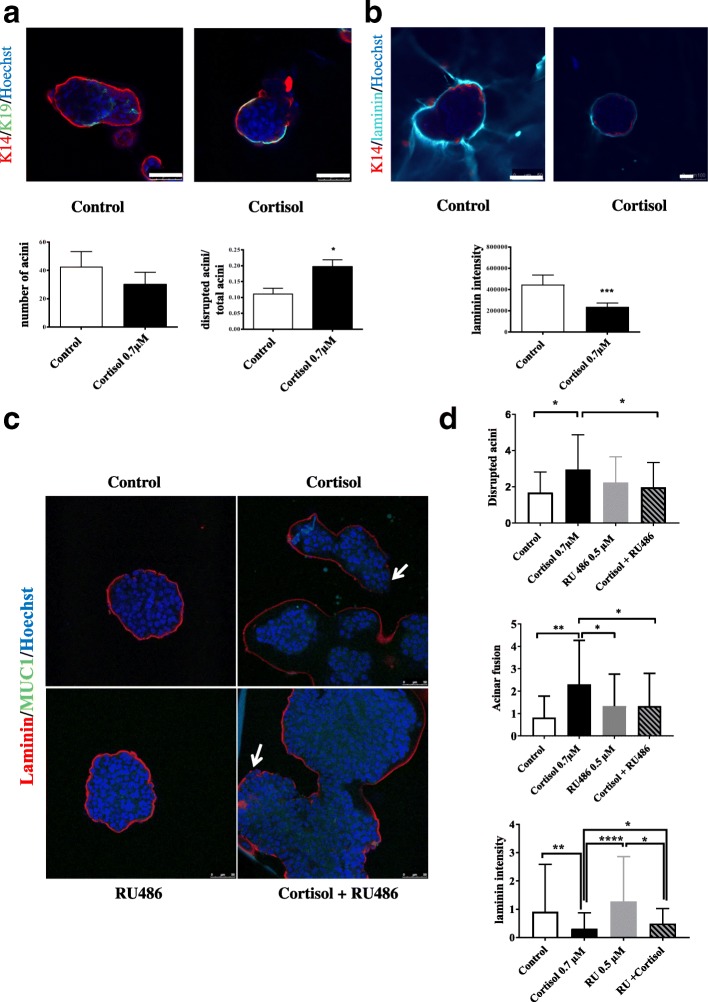
Influences of cortisol treatment on 3D growth of mammary epithelial cells for 14 days identified by immunofluorescence. Cells were treated with cortisol 0.7 μM or vehicle (methanol) from day 5 after seeding until day 14. **a** Upper panels: immunodetection in primary epithelial and myoepithelial cells of K14 (myoepithelial cells), K19 (epithelial cells) and Hoechst dye was used as *nuclei* counterstaining. Scale bar = 50 μm. Bottom panels: quantification of morphometric analysis in the control group and cortisol-treated group of the number of *acini* formed and quantification of disrupted *acini* per total number of *acini*. **b** Upper panels: immunofluorescence of laminin (BM), cytokeratin14 (myoepithelial cells) and Hoechst dye to counterstain *nuclei*. Scale bar = 100 μm. Bottom panels: quantification by Image J software of laminin intensity after cortisol 0.7 μM or vehicle treatment. **c** Immunofluorescence in MCF10DCIS 3D growth of laminin (basement membrane), Muc1 (epithelial cells) and Hoechst dye to stain the *nuclei* under control (vehicle), cortisol 0.7 μM, RU486 0.5 μM and cortisol 0.7 μM + RU486 0.5 μM. Arrows indicate rupture points of the *acini*. **d** Morphometric quantification of disrupted *acini* and acinar fusion and intensity of laminin determined by the integrated density parameter of Image J software. Scale bar = 50 μm. All experiments were carried out in triplicate. Statistical analysis was performed using the Mann-Whitney test or one-way analysis of variance followed by Tukey post-hoc test

We seeded the cells on matrigel and let the *acini* grow for 5 days before treating them with cortisol for 9 days. Subsequently, we searched for epithelial cell (cytokeratin 19) and myoepithelial cell (cytokeratin 14) marker expression. We found that cortisol affected the capacity of epithelial and myoepithelial mammary cells to form acinar structures because the number of *acini* was smaller than in untreated cell cultures (Fig. 2a). In addition, cortisol treatment seems to disrupt the myoepithelial cell layer, as the number of disrupted *acini *increased significantly (Fig. 2a). To further study myoepithelial cell layer disruption, we also stained the 3D structures for laminin, the principal component of the BM, which is usually produced by myoepithelial cells [10]. We found that, when treated with cortisol, the amount of laminin surrounding the *acini* was significantly reduced (Fig. 2b). This suggests that when treated with cortisol, the *acini* acquire a more invasive phenotype and that apoptosis or cell cycle arrest of myoepithelial cells might in part be responsible for myoepithelial layer disruption (Fig. 2a-b). These results are in agreement with our 2D results, since apoptosis of myoepithelial cells caused by cortisol may affect their capacity to form duct-like structures and would favour invasion by disrupting the myoepithelial cell layer.

We made 3D cultures of MCF10DCIS cells and treated them with cortisol to determine whether this molecule has the same effect on MCF10DCIS cells, which were resistant to cortisol in MTT experiments (Fig. 1a, Fig. 2c-d). After treatment with cortisol, laminin intensity decreased and the proportion of acinar fusion increased (Fig. 2c-d), suggesting that treatment with cortisol also promotes the invasive capacity of DCIS acinar-like structures. Additionally, treatment with the GR antagonist, RU486, was able to block the effects of cortisol on the functional abilities of the MCF10DCIS 3D cultures (Fig. 2c-d). In fact, it prevented cortisol induction of acinar fusion and disruption (Fig. 2c-d). However, RU486 treatment only partially prevented cortisol inhibition of laminin levels (Fig. 2c-d). These results suggest that cortisol via its receptor GR is responsible for the acceleration of MCF10 DCIS acquisition of invasive features.

### Glucocorticoids accelerate the progression of DCIS to IDC in vivo by inducing myoepithelial cell layer disruption

To better understand the role of myoepithelial cells in the progression of breast cancer and to study the dynamics of the formation and disruption of the myoepithelial layer under stress, we established an in vivo model using immunosuppressed mice and the MCF10DCIS cell line, which generates DCIS when inoculated into immunosuppressed mice and then evolves to IDC [18]. First, we established the dynamics of the formation and disruption of the myoepithelial cell layer in a control situation (Additional file 5: Figure S2A). We inoculated 10^5^ cells in the mammary fat pad of mice that were subsequently killed at different times (Additional file 5: Figure S2A). Tumours were fixed and stored as paraffin-embedded tissue to study the tumour histology with haematoxylin and eosin staining (H&E) and by immunohistochemical analysis using antibodies against the myoepithelial cell markers alpha-smooth muscle actin (αSMA) and p63 (Additional file 5: Figure S2A).

The results showed that the first duct-like structures, surrounded by a layer of cells positive for αSMA and p63 (Additional file 5: Figure S2A), were formed at day 7 after inoculation of MCF10DCIS cells. At day 22, the myoepithelial cell layer was widely ruptured and by day 29 duct organization was virtually lost. At this point, some αSMA-positive cells were tumour-associated fibroblasts, while a subset of tumour epithelial cells was p63 positive (Additional file 5: Figure S2A). To investigate whether MCF10DCIS epithelial or myoepithelial cells could respond to glucocorticoids in vivo, we analysed the expression of GR in these tumours and found that both epithelial and myoepithelial cells expressed GRs (Additional file 5: Figure S2B).

Since corticosterone is the glucocorticoid expressed in mice, we determined whether it had the same effects as cortisol (Additional file 6: Figure S3) and found that it inhibited proliferation in myoepithelial cells and to a lesser extent in MCF10A cells while MCF10DCIS cells were also resistant to corticosterone (Additional file 6: Figure S3A). Furthermore, similar to cortisol, treatment with corticosterone induced apoptosis only in myoepithelial cells (Additional file 6: Figure S3B). Moreover, corticosterone treatment also induced the disruption of 3D culture *acini* and inhibited laminin expression in a co-culture of primary epithelial and myoepithelial cells and also in the MCF10DCIS cell line (Additional file 6: Figure S3C-F). These results suggest that corticosterone might promote invasiveness through myoepithelial cell apoptosis, as did cortisol.

**Fig. 3:**
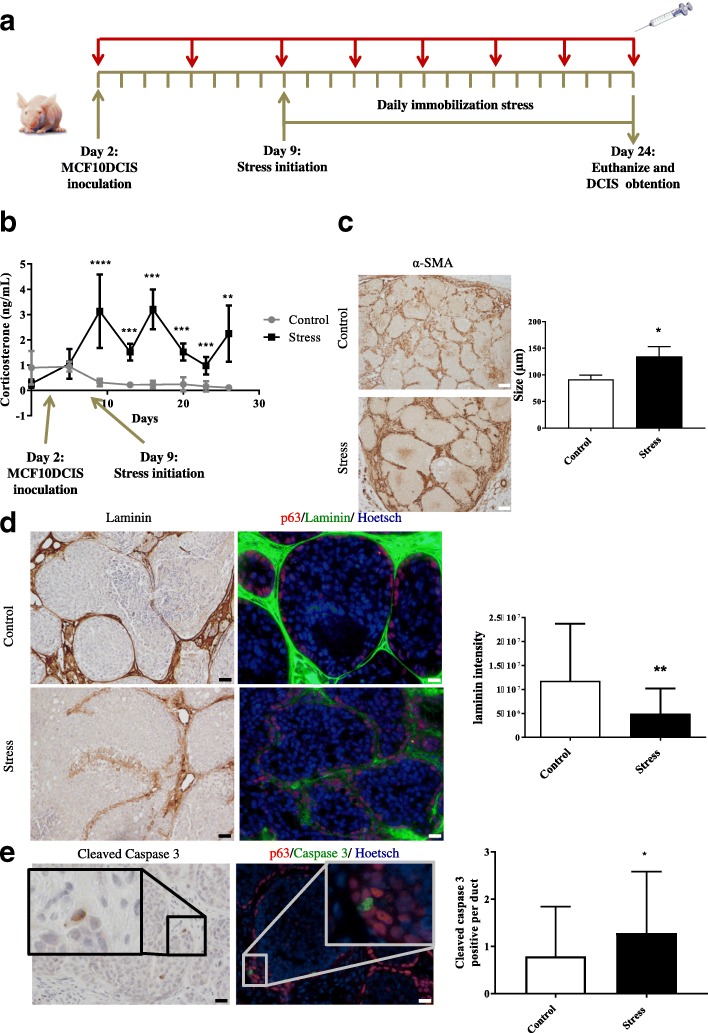
In vivo stress model and the effects on evolution of the MCF10DCIS xenograft. **a** Timeline of the in vivo stress model indicating blood extraction and the immobilization method applied. **b** Corticosterone levels (ng/ml) in plasma samples obtained at different points of the in vivo stress model in mice, in the control and stressed group of MCF10DCIS xenograft. The Wilcoxon paired test was used for statistical analysis. **c** Left panel: representative image of α-smooth muscle actin (α-SMA, a myoepithelial cell marker) from immunohistochemical analysis of the control and stressed tumours in mice. Scale bar = 50 μm. **c** Right panel: duct size quantification by Image J Software. In vivo experiments were performed using five animals per group. The Mann-Whitney test was used for statistical analysis. **d** Left panel: representative laminin immunohistochemical images in control and stressed MCF10DCIS xenografts. Scale bar = 50 μm. **d** Right panel: laminin and p63 double immunofluorescence and quantification of laminin intensity images (Image J Software) of tumours derived from control and stressed mice. Hoechst dye was used to counterstain the *nuclei*. Scale bar = 20 μm. **e** Left panel: representative cleaved caspase 3 immunohistochemical image of a tumour from stressed mice. Scale bar = 20 μm. **e** Middle panel: representative cleaved caspase 3(green) and p63(red) immunofluorescence image of a tumour from stressed mice. Hoechst dye was used as the counterstain for *nuclei.* Scale bar = 20 μm. **e** Right panel: quantification of caspase 3-positive myoepithelial cells per duct in control and tumours from stressed mice; *n* = 5 animals/group. The Mann-Whitney test was used for statistical analysis. DCIS, ductal carcinoma in situ

To determine whether glucocorticoids promote DCIS transition to IDC, MCF10DCIS cells were inoculated into mice mammary fat pads, and 9 days later when DCIS was established immobilization stress was initiated to mimic chronic stress (Fig. 3a). Blood was extracted from mice tails every 3 days and corticosterone levels were measured using ELISA (Fig. 3a-b). We have previously found that in vitro corticosterone treatment of MCF10DCIS cells produces similar effects to cortisol on cell viability, apoptosis and functional 3D abilities (Fig. 2c-d and Additional file 6: Figure S3A-B), favouring the breakdown of the myoepithelial cell layer and the disappearance of the BM in a similar fashion to cortisol.

Mice underwent stress daily until they were killed at 24 days after inoculation, when the myoepithelial cell layer was mostly ruptured (Fig. 3c). Tumours were extracted, fixed and stored as paraffin-embedded tissue. As expected, corticosterone levels significantly increased when stress began and remained elevated throughout the experiment (Fig. 3b).

To determine how the architecture of duct-like structures changed in control and stressed mice tumours, histology was examined by H&E staining and immunohistochemical analysis was performed using antibodies against the myoepithelial cell and basement membrane markers α-SMA and laminin (Fig. 3c-d). We found that although the ducts in both control and stressed tumours were starting to rupture, there was greater disorganization of the ductal architecture when mice underwent stress (Fig. 3c-d), and the ducts were significantly larger in the tumours of stressed animals, suggesting they may have been disrupted earlier, joining together and forming larger ductal structures (Fig. 3c-d).

To determine how stress affected the invasiveness of DCIS, BM loss was also analysed by laminin staining, a key feature of invasiveness. Laminin immunohistochemical and double immunofluorescence assays, together with p63 staining, were used and the results showed stressed mice had significantly less laminin surrounding the ducts (Fig. 3d), thus showing a more invasive phenotype.

As the treatment of myoepithelial cell cultures with corticosterone in vitro caused apoptosis (Additional file 6: Figure S3B), we analysed the number of apoptotic myoepithelial cells per duct and found more apoptotic myoepithelial cells in tumours from stressed mice, who were thus exposed to higher corticosterone levels (Fig. 3e).

These results may suggest that chronic stress through sustained high blood corticosterone levels and other mechanisms promotes myoepithelial cell apoptosis, leading to disruption of the myoepithelial cell layer, favouring cell invasion and fostering progression from DCIS to IDC.

**Fig. 4:**
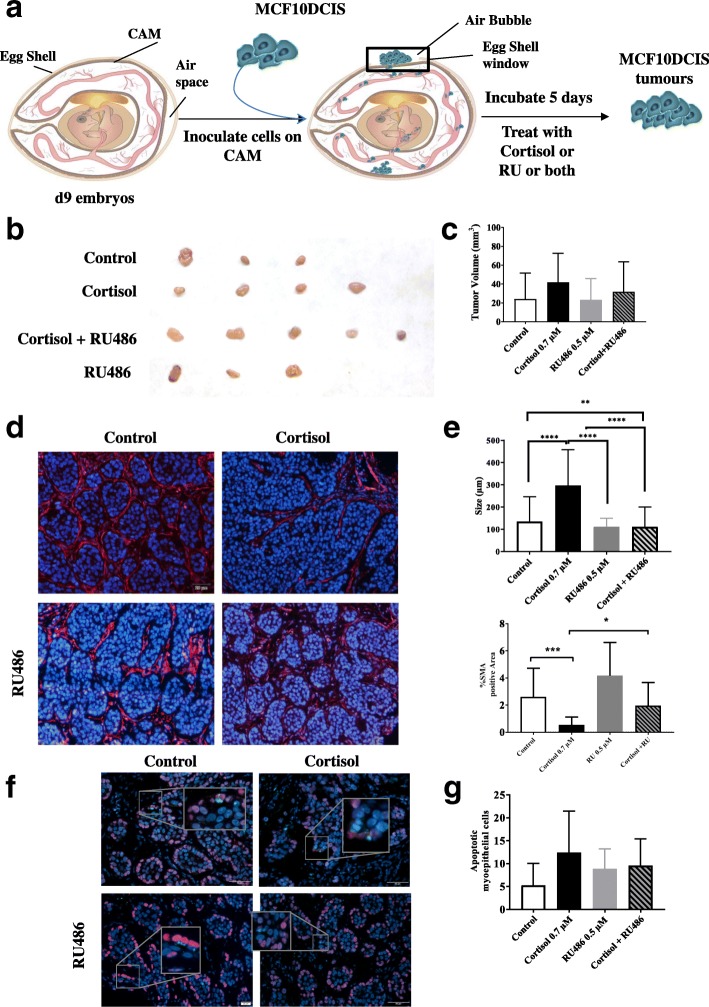
In vivo chicken embryo chorioallantoic membrane (CAM) system for growing MCF10DCIS tumours. **a** Timeline of CAM model indicating how the inoculation of MCF10DCIS cells was performed and how the tumours were treated. **b** Representative tumour images after treating with control, cortisol 0.7 μM, RU486 0.5 μM and cortisol + RU486 for 5 days. **c** Graphical representation of tumour volume in mm^3^. **d** α-Smooth muscle actin (α-SMA) (in red) immunofluorescence of the tumours after treatment with cortisol 0.7 μM, RU486 0.5 μM and cortisol + RU486. Hoechst dye was used to counterstain *nuclei*. Scale bar =20 μm. **e** Quantification of *acini* size (μm). Bottom panel: representation of percentage of α-SMA-positive area in tumours. Scale bar = 50 μM. **f** Images of double p63 (myoepithelial cells) and cleaved caspase 3 immunofluorescence in tumours. Scale bar = 50 μM**. g** Graphical representation of apoptotic myoepithelial cells quantification in tumours under the different treatments. One-way analysis of variance was used for statistical analysis followed by Tukey post-hoc test

Additionally, we used the chick embryo chorioallantoic membrane (CAM) system for growing MCF10DCIS tumours (Fig. 4a). First of all, we tested whether when inoculated in CAM, MCF10DCIS form the same structures as they do in xenografts on mice (Additional file 7: Figure S4). The double p63 and laminin immunofluorescence carried out in the tumour sections showed typical organization of MCF10DCIS tumour growth and a proper laminin and p63 distribution (Additional file 7: Figure S4). Thus, we have used this model to study the effects of cortisol and the GR antagonist, RU486, on the transition of MCF10DCIS tumours to IDC in vivo (Fig. 4). Our results have shown that cortisol treatment slightly increases tumour volume, while treatment with cortisol and RU486 partially reverts this effect. In a similar way, treatment with cortisol induces acinar fusion and increases *acini* size (Fig. 4d-e). Furthermore, the myoepithelial cell layer detected by staining of αSMA positive cells was reduced after cortisol treatment (Fig. 4f). Interestingly, treatment with GC antagonist alone slightly increases αSMA staining. However, combination of cortisol and RU486, not only reverted cortisol inhibition of αSMA and disruption of the myoepithelial cell layer, but also prevented cortisol induction of acinar fusion and reduced *acini* size (Fig. 4d-f).

We also quantified myoepithelial cell apoptosis (Fig. 4g-h). In agreement with our previous in vitro results, the number of apoptotic myoepithelial cells was increased after treatment with cortisol. Interestingly, RU486 treatment partially reverted the effects of cortisol on myoepithelial cell apoptosis (Fig. 4g-h). Thus, treatment with RU486 in vivo hampers cortisol induction of acinar fusion, degradation of the BM and myoepithelial cells apoptosis, preventing cortisol promotion of DCIS invasiveness.

### Myoepithelial cell fate in patients with DCIS and DCIS+IDC

**Fig. 5:**
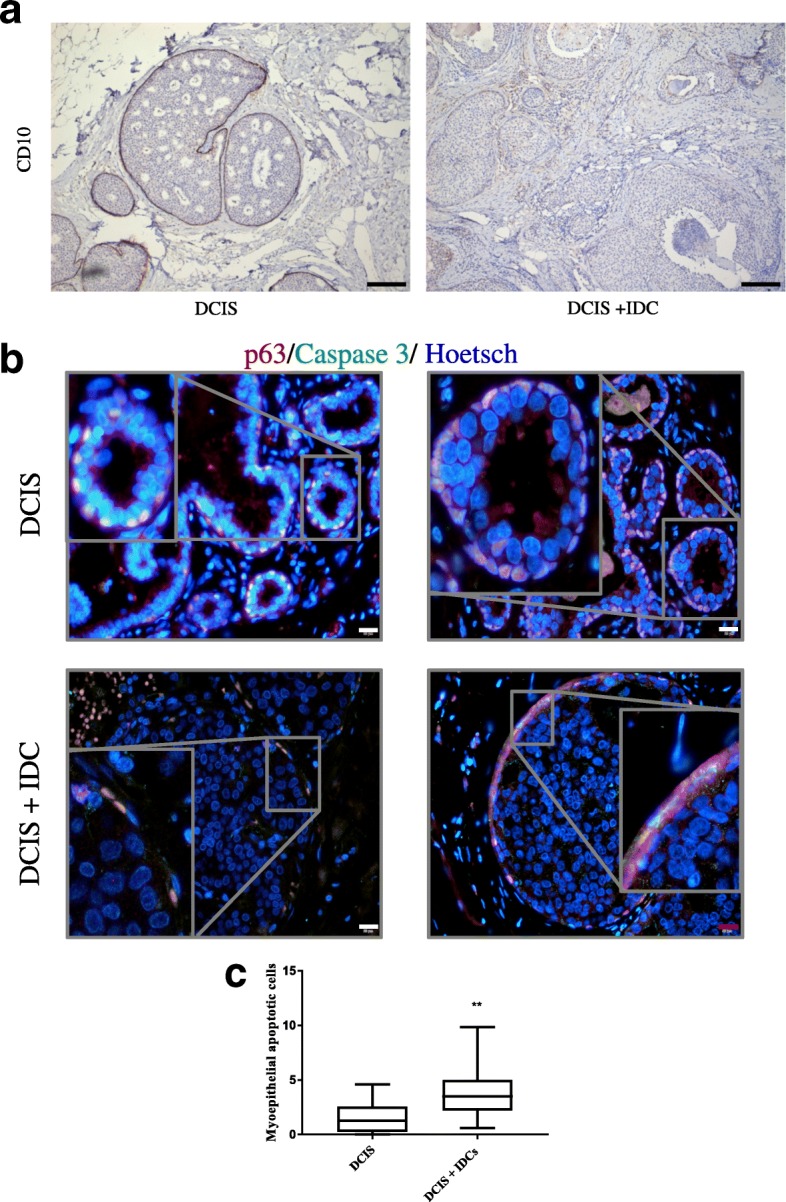
Immunohistochemical and immunofluorescence images of human samples from patients with ductal carcinoma in situ (DCIS) and DCIS + invasive ductal carcinoma (IDC). **a** CD10 (myoepithelial cells) immunohistochemical analysis of samples from patients. Scale bar = 100 μm. **b** Double immunofluorescence of p63 (myoepithelial cells) and cleaved caspase 3 and Hoechst dye as a *nuclei* counterstain. Upper panel: patients with DCIS; bottom panel: patients with DCIS + IDC. White scale bar = 20 μm and red scale bar = 50 μm. **c** Quantification of apoptotic myoepithelial cells in DCIS (13 patients) and DCIS + IDC (15 patients). The Mann-Whitney test was used for statistical analysis

**Fig. 6:**
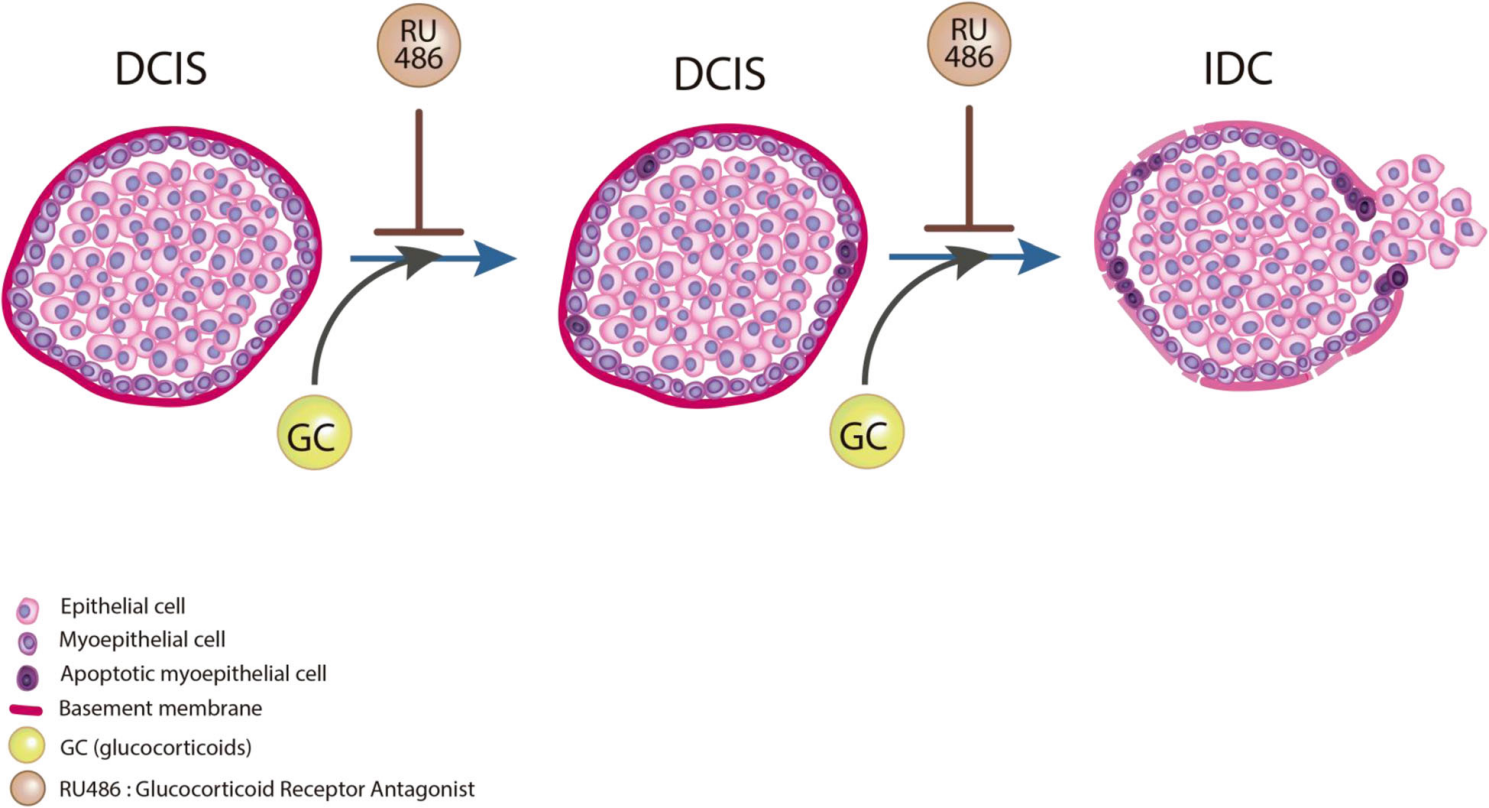
Effects of glucocorticoids (GC) on ductal carcinoma in situ (DCIS) transition to invasive ductal carcinoma (IDC). Glucocorticoids promote progression of DCIS to invasiveness by reducing laminin levels and inducing myoepithelial cell apoptosis in vitro and in vivo*,* effects that can be partially blocked by the glucocorticoid receptor antagonist RU486

As we have found that myoepithelial cell apoptosis contributes to disruption of the myoepithelial cell layer and the BM and promotes the invasiveness of DCIS, we wondered if patients with DCIS+IDC would have more apoptotic myoepithelial cells in the ducts, indicating a more invasive DCIS phenotype. Therefore, we obtained breast tissue samples from patients with DCIS or DCIS+IDC and evaluated myoepithelial cell apoptosis. We analysed tissue samples from 13 patients with DCIS and 15 patients with DCIS+IDC (Additional file 3: Table S2) with both immunohistochemistry against CD10 (myoepithelial cells) (Fig. 5a) and double immunofluorescence against p63 (myoepithelial cells) and cleaved caspase 3 (apoptosis marker) (Fig. 5b-c and Additional file 8: Figure S5). The results showed that patients with DCIS+IDC lost most of the continuous p63 staining (Fig. 5b bottom part, 5C and Additional file 8: Figure S5) and exhibited myoepithelial cell apoptosis in some ducts, whereas patients with DCIS did not present apoptosis of myoepithelial cells in most ducts, although isolated apoptotic events were detected (Fig. 5b upper part, 5C and Additional file 8: Figure S5), suggesting myoepithelial cell apoptosis is a prerequisite for DCIS invasiveness (Fig. 6) and that the use of RU486 could partially prevent this event.

## Discussion

Understanding the mechanisms that initiate and trigger DCIS invasiveness is relevant because understanding how DCIS evolves into invasive carcinoma could enable the design of interventional strategies to prevent breast cancer progression. The most widely accepted hypothesis on the mechanisms regulating the transition from DCIS to IDC suggests the microenvironment is involved, in particular fibroblasts and myoepithelial cells [14, 15, 43]. Despite recent progress in myoepithelial cell research suggesting that they may act as tumour suppressors [11], the mechanism of myoepithelial cell layer disruption remains elusive.

In this scenario, the present study investigated the role of glucocorticoids in the transition of DCIS to invasiveness. The effect of stress-related factors, studied as microenvironmental elements, in the progression of breast cancer has been investigated [18, 44]. However, the impact of glucocorticoids in the transition from DCIS to IDC has not yet been studied. To the best of our knowledge, this is the first study to suggest that glucocorticoids contribute to the transition from DCIS to IDC, particularly through the disappearance of the basal lamina and the induction of myoepithelial cell apoptosis. In addition, myoepithelial cell apoptosis is prominent in breast cancer tumours that have progressed to IDC, suggesting apoptosis could be a preliminary, essential factor that precedes invasiveness.

Glucocorticoids have been shown in studies to mediate growth inhibition in some mammary tumour cell lines [45], and to induce G1/G0 cell cycle arrest [46], and if administration persists, to lead to cell apoptosis [47]. However, there are contrasting results on the effect of glucocorticoids on cell survival and proliferation [45, 48]. For instance, in breast invasive tumours, glucocorticoids promote cell survival through the induction of anti-apoptotic genes [29]. In contrast, glucocorticoids trigger apoptosis in hematopoietic cells and lymphocytes [28], suggesting they may act differently depending on the cell subtype. GRs are expressed in luminal epithelial cells and occasionally in stromal cells, but predominantly in myoepithelial cells [30], suggesting that GR are expressed in a cell-lineage-dependent manner in breast tissue [30] as reported in other tissues [45, 46], and that myoepithelial cells may play a physiological role in mediating the effects of glucocorticoid hormones in the breast.

Furthermore, our results showed that glucocorticoids induce the degradation of the BM and the disruption of acinar structures through a significant loss of laminin in healthy human mammary epithelial and myoepithelial cells and in MCF10DCIS 3D cultures. Laminin is a prominent and influential component of the BM, which constitutes a physical barrier for invasive epithelial cells [49–51]. Laminin expression and αSMA levels are both related to tumour malignancy [52, 53]. Similarly, our in vivo stress model and our in vivo chicken embryo CAM system showed indicators of higher invasiveness after stress challenge [10], in particular increased acinar rupture and reduced laminin levels or αSMA levels. Moreover, treatment of MCF10DCIS 3D cultures with the GR antagonist, RU486, blocked the effects of cortisol on the integrity of the acinar structures and also on the BM, highlighting the influence of cortisol on the early acquisition of invasive features in breast cancer cells. We hypothesize that the loss of laminin is directly related to myoepithelial cell loss by apoptosis. However, more experiments are needed to confirm this idea.

In this context, repeated immobilization has been described as a model of chronic stress since in our case it was applied for 2 h every day for a period of 4 weeks. In addition, the repeated immobilization model is reported to increase corticosterone plasma levels among other changes [54]. In fact, chronic stress situations initiate a cascade of pathways in the central nervous system and periphery, triggering fight-or-flight stress responses in the autonomic nervous system (ANS) or defeat/withdrawal responses in the hypothalamic-pituitary-adrenal axis (HPA) [55]. ANS responses to stress are mediated by the activation of the sympathetic nervous system (SNS) and the following release of cathecolamines [56]. HPA responses to stress include hypothalamic production of corticotrophin-releasing hormone (CRH) and arginine vasopressin (AVP). These molecules activate the secretion of pituitary hormones and adrenocorticotropic hormones (ACTH), which induces the release of glucocorticoids from the adrenal cortex [55]. In this scenario, glucocorticoids are the final effectors of the HPA axis, which regulate CRH and ACTH secretion and limit the duration of the total tissue exposure of the organism to glucocorticoids [57].

In agreement with our findings connecting glucocorticoids with the invasiveness of DCIS, several studies have linked glucocorticoids to the progression and malignancy of breast cancer. In animal models, glucocorticoid regulation following exposure to social isolation was associated with an increased mammary tumour burden [28], while acute stressors were linked to abnormal glucocorticoid regulation and increased mammary tumour growth [58]. Pan et al. identified correlation between the expression of GRs in estrogen receptor (ER)^−^ breast tumours with shorter relapse-free survival [59]. In addition, women with metastatic breast cancer frequently have flatter-than-normal diurnal cortisol patterns and the degree of diurnal variation of glucocorticoids may predict earlier breast cancer mortality [60]. Glucocorticoids have also been linked to reduced immunosurveillance, which is associated with the induction of tumour progression [61, 62]. These studies and our results suggest that a possible side effect of glucocorticoid therapy would be a higher risk of evolution to invasive breast cancer in patients who might have preneoplastic lesions that have not been diagnosed.

After stress challenge or glucocorticoid treatment, apoptotic myoepithelial cells were more frequent in patients with DCIS+IDC than in patients with DCIS, in agreement with a study that described apoptosis in the myoepithelial cell layer in comedo-type DCIS [13]. The authors claimed that it was an early event associated with the central necrosis of this type of DCIS, both in MCF10DCIS xenografts and patient samples [13]. Samples from our patients with DCIS+IDC seemed to display more malignant characteristics than those from patients with DCIS. Since the apoptotic myoepithelial cells are in the intact DCIS, this suggests that myoepithelial cell apoptosis might be a prerequisite for disruption of the myoepithelium and the consequent invasion of the epithelial compartment into the stroma.

## Conclusions

In conclusion, we found that chronic stress, through sustained glucocorticoid treatment, plays a role in the progression of DCIS to invasiveness, particularly by promoting myoepithelial cell apoptosis in vitro and in vivo. Myoepithelial cell apoptosis differentiates patients with DCIS from those with DCIS+IDC, and might be a prerequisite for invasive features. Thus, the use of glucocorticoid inhibitors or other therapies that might prevent myoepithelial cell apoptosis could potentially interfere with and prevent the progression of DCIS to invasiveness. In addition, the prevalence of apoptosis in myoepithelial cells in DCIS samples might be used as a prognostic factor for progression to IDC.

## Additional file

Additional file 1: **Table S1.** Description of patient samples used for the primary mammary epithelial and myoepithelial cells. Internal codes, histological description and age of patients. (PPTX 53 kb)

Additional file 2: Tissue digestion and cell fraction separation protocol. (PDF 231 kb)

Additional file 3: **Table S2.** Description of patient samples used to confirm apoptosis in human myoepithelial cell samples. Internal codes, histopathological description and receptor characteristics of patient samples. ND: not detectable. NA: not applicable. A: amplified. (PPTX 90 kb)

Additional file 4: **Figure S1.** Immunofluorescence of glucocorticoid receptor in myoepithelial, MCF10A and MCF10DCIS cells. Hoechst was used to counterstain nuclei. Scale bar= 20 µm. (PPTX 917 kb)

Additional file 5: **Figure S2.** In vivo progression of MCF10DCIS xenografts. **a** Histology of tumours (H&E) and expression of α-SMA and p63 were analysed at the indicated time points after injection (n=5 animals). Scale bar= 100 µm. **b** Representative image of glucocorticoid receptor immunohistochemistry in in vivo samples. Scale bar= 20 µm. Red arrow indicates myoepithelial positive cells for GR and black arrow shows positivity in epithelial cells. (PPTX 5642 kb)

Additional file 6: **Figure S3.** Corticosterone effects on mammary epithelial cells viability, apoptosis and functional abilities. **a** MCF10A epithelial, primary myoepithelial cells and MCF10DCIS cell viability evaluated by MTT assay after 48h of increasing doses of corticosterone treatment (0.125-1.5 µM). **b** MCF10A epithelial, primary myoepithelial cells and MCF10DCIS determination of cell apoptosis in its different stages (early, apoptosis, late and total) after treatment with corticosterone 0-1 µM by the Annexin V method and measured by flow cytometry. All experiments were carried out in triplicate. Statistical analysis was made using ANOVA followed by Dunn’s multiple test. **c** and **d**. Influence of corticosterone treatment on 3D growth of primary epithelial and myoepithelial cells and on MCF10DCIS for 14 days by immunofluorescence. Treatment with corticosterone 1 µM or vehicle (methanol) was carried out from day 5 after seeding until day 14. **c **Upper part. Immunodetection of K14 (myoepithelial cells), K19 (epithelial cells), with hoechst used as *nuclei* counterstaining. Scale bar=50 µm. **c** Bottom part. Quantification of morphometric analysis in control group and corticosterone-treated group of number of *acini* formed and related quantification of disrupted *acini* per total number of *acini*.** d** Upper part. Immunofluorescence of laminin (basement membrane), K14 (myoepithelial cells) and hoechst to counterstain *nuclei*. Scale bar=100 µm. **d** Bottom part. Quantification of laminin intensity after treatment with corticosterone 1 µM or vehicle by Image J software comparison test. **e** Immunofluorescence in MCF10DCIS 3D growth of laminin (basement membrane), Muc1 (epithelial cells) and hoechst to stain the *nuclei*. Arrows indicated rupture points of the *acini* showed. F. Morphometric quantification of disrupted *acini* and acinar fusion and intensity of laminin determined by integrated density parameter of Image J software. Scale bar=50µm. All experiments were carried out in triplicate. Statistical analysis was made using the Mann-Whitney test. (PPTX 3174 kb)

Additional file 7: **Figure S4.** Representative immunofluorescence images of MCF10DCIS xenografts in chicken embryo CAM membrane.**a **Hoechst in blue, p63 in red and laminin in green.** b** Merge of p63 and laminin double immunofluorescence images and zoom in showing an *acini* detail. Scale bar=20 µm. (PPT 4617 kb)

Additional file 8: **Figure S5.** Representative immunofluorescence images of human samples of DCIS (6 patients) and DCIS + IDC (6 patients).**a** Double immunofluorescence of p63 (myoepithelial cells) and cleaved caspase 3 indicated with red arrows and hoechst as a *nuclei* counterstainer in DCIS sample patients and **b** in DCIS + IDC sample patients. White scale bar=20 and red scale bar=50 µm. (PPTX 13532 kb)

## Abbreviations

ACTH: Adrenocorticotropic hormone
ANS: Autonomic nervous system
α-SMA: α-Smooth muscle actin
3D: Three-dimensional
BM: Basement membrane
BrdU: Bromodeoxyuridine
CAM: Chorioallantoic membrane
CRH: Corticotrophin-releasing hormone
DCIS: Ductal carcinoma in situ
ELISA: Enzyme-linked immunosorbent assay
FITC: Fluorescein isothiocyanate
GR: Glucocorticoid receptor
HPA: Hypothalamic-pituitary-adrenal axis
HRP: Horseradish-peroxidase conjugated
IDC: Invasive ductal carcinoma
IDIBAPS: Institut d’Investigacions Biomèdiques August Pi i Sunyer
MTT: 3-(4, 5-Dimethylthiazol-2-yl)-2, 5-diphenyl tetrazolium bromide
PBS: Phosphate-buffered saline
PI: Propidium iodide
RT: Room temperature
