# Supplementary material for: Glucocorticoids promote transition of ductal carcinoma in situ to invasive ductal carcinoma by inducing myoepithelial cell apoptosis
Source: Breast Cancer Res. 2018 Jul 4;20:65. doi: 10.1186/s13058-018-0977-z (PMC6032539; doi:10.1186/s13058-018-0977-z)
Supplement: Supplementary file 1 — Table S1. Description of patient samples used for the primary mammary epithelial and myoepithelial cells. Internal codes, histological description and age of patients. (PPTX 53 kb) [file 13058_2018_977_MOESM1_ESM.pptx]

## Slide 1
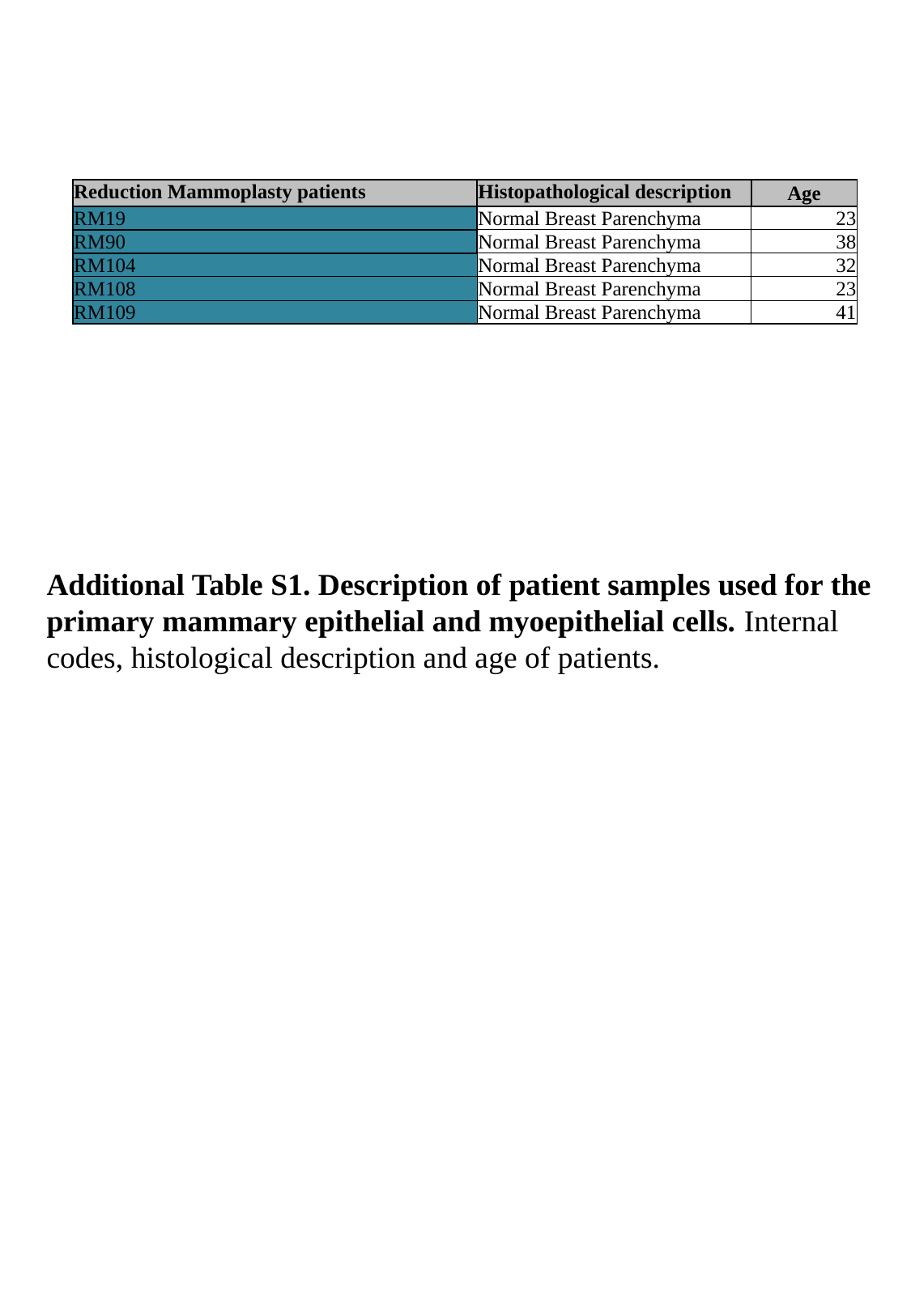

| Reduction Mammoplasty patients | Histopathological description | Age |
| --- | --- | --- |
| RM19 | Normal Breast Parenchyma | 23 |
| RM90 | Normal Breast Parenchyma | 38 |
| RM104 | Normal Breast Parenchyma | 32 |
| RM108 | Normal Breast Parenchyma | 23 |
| RM109 | Normal Breast Parenchyma | 41 |
Additional Table S1. Description of patient samples used for the primary mammary epithelial and myoepithelial cells. Internal codes, histological description and age of patients.
