# Supplementary material for: Glucocorticoids promote transition of ductal carcinoma in situ to invasive ductal carcinoma by inducing myoepithelial cell apoptosis
Source: Breast Cancer Res. 2018 Jul 4;20:65. doi: 10.1186/s13058-018-0977-z (PMC6032539; doi:10.1186/s13058-018-0977-z)
Supplement: Supplementary file 2 — Tissue digestion and cell fraction separation protocol. (PDF 231 kb) [file 13058_2018_977_MOESM2_ESM.pdf]

Tissue digestion and cell fraction separation

Samples were obtained after approval by the Institutional Review Board of the Hospital Clinic and Clinica Planas, Barcelona. Between 40 and 80 g of fresh mammary tissue from five women aged 20-45 years was used as starting material (**Supplementary Table 1**). Briefly, the material was minced into small pieces and digested at 37ºC overnight (ON) in a shaker in DMEMF12 medium with 200 U/mL of collagenase type IV (Sigma, MO, USA) and 100 U/mL of hyaluronidase (Sigma, MO, USA), as we previously described (1). Subsequently, organoids, single epithelial cells and stromal cells were separated by differential centrifugation at 40 g for 1 min, 100 g for 2 min and 200 g for 4 min, respectively.

Bibliography

1. Zubeldia-Plazaola A, Ametller E, Mancino M*, et al.* Comparison of methods for the isolation of human breast epithelial and myoepithelial cells. Front Cell Dev Biol 2015;3: 32.
