# Supplementary material for: Glucocorticoids promote transition of ductal carcinoma in situ to invasive ductal carcinoma by inducing myoepithelial cell apoptosis
Source: Breast Cancer Res. 2018 Jul 4;20:65. doi: 10.1186/s13058-018-0977-z (PMC6032539; doi:10.1186/s13058-018-0977-z)
Supplement: Supplementary file 4 — Figure S1. Immunofluorescence of glucocorticoid receptor in myoepithelial, MCF10A and MCF10DCIS cells. Hoechst was used to counterstain nuclei. Scale bar= 20 µm. (PPTX 917 kb) [file 13058_2018_977_MOESM4_ESM.pptx]

## Slide 1
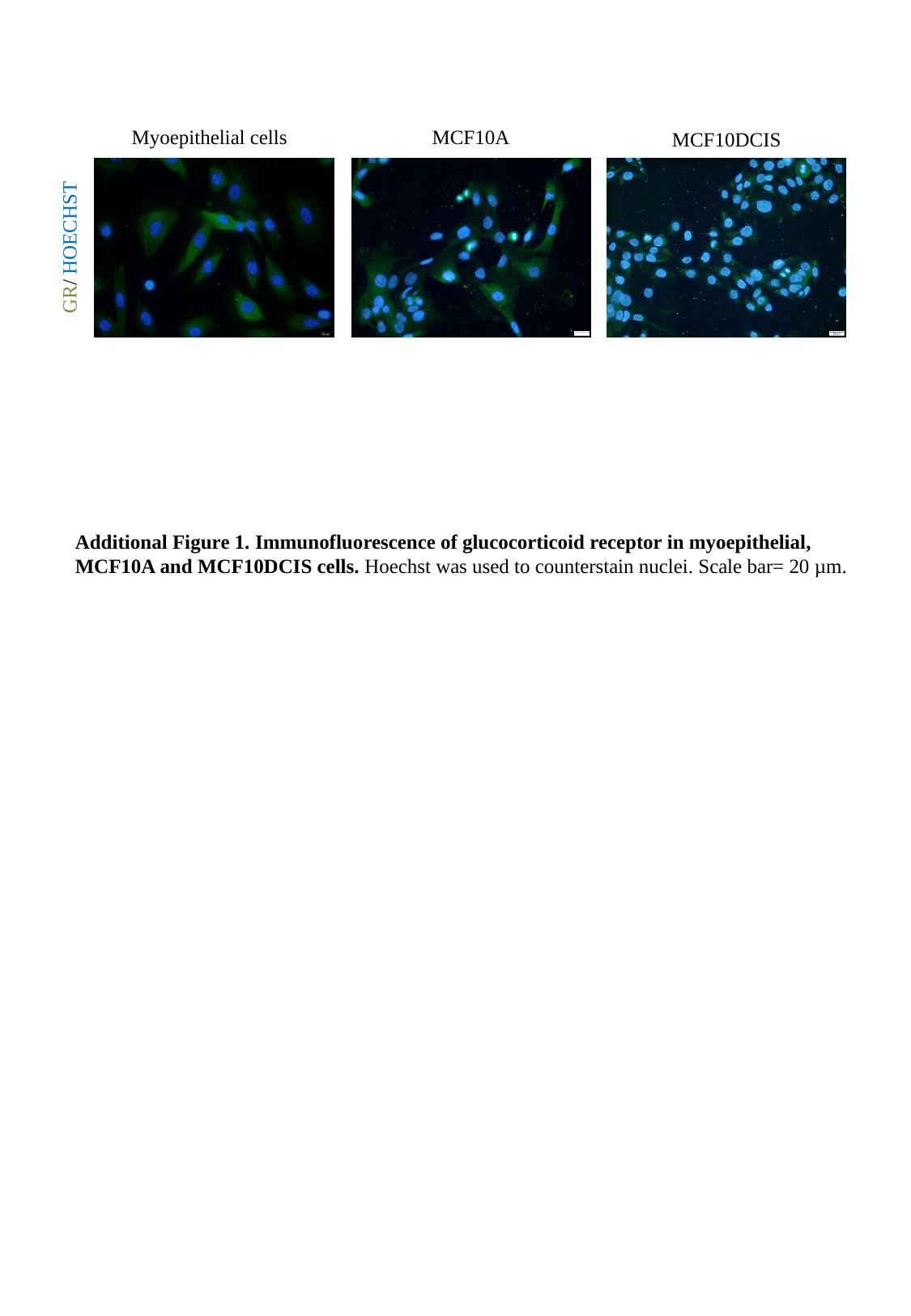

Myoepithelial cells
MCF10A
MCF10DCIS
GR/ HOECHST
Additional Figure 1. Immunofluorescence of glucocorticoid receptor in myoepithelial, MCF10A and MCF10DCIS cells. Hoechst was used to counterstain nuclei. Scale bar= 20 µm.
