# Supplementary material for: Glucocorticoids promote transition of ductal carcinoma in situ to invasive ductal carcinoma by inducing myoepithelial cell apoptosis
Source: Breast Cancer Res. 2018 Jul 4;20:65. doi: 10.1186/s13058-018-0977-z (PMC6032539; doi:10.1186/s13058-018-0977-z)
Supplement: Supplementary file 5 — Figure S2. In vivo progression of MCF10DCIS xenografts. a Histology of tumours (H&E) and expression of α-SMA and p63 were analysed at the indicated time points after injection (n=5 animals). Scale bar= 100 µm. b Representative image of glucocorticoid receptor immunohistochemistry in in vivo samples. Scale bar= 20 µm. Red arrow indicates myoepithelial positive cells for GR and black arrow shows positivity in epithelial cells. (PPTX 5642 kb) [file 13058_2018_977_MOESM5_ESM.pptx]

## Slide 1
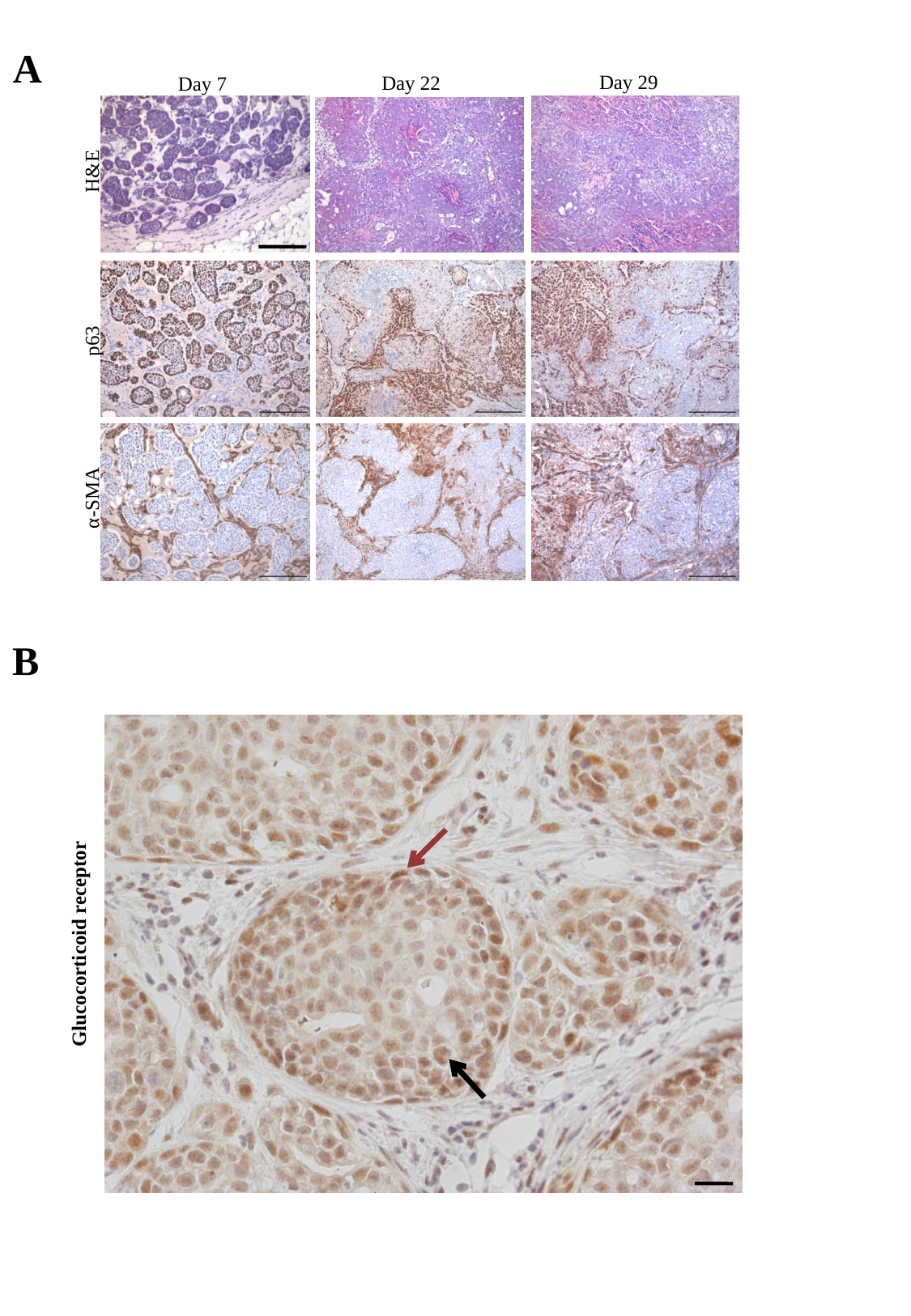

A
Day 29
Day 22
Day 7
H&E
p63
α-SMA
B
Glucocorticoid receptor

## Slide 2
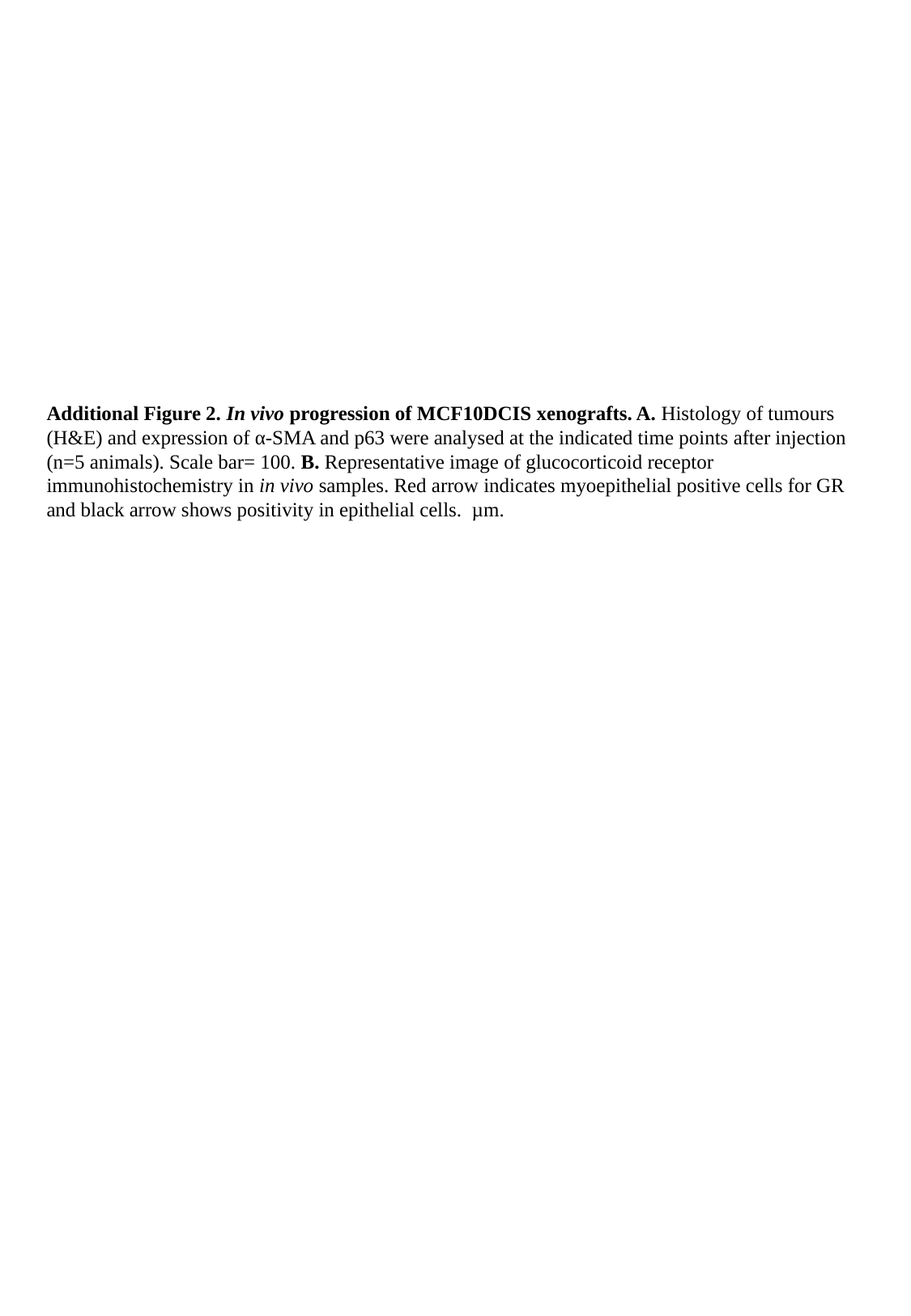

Additional Figure 2. In vivo progression of MCF10DCIS xenografts. A. Histology of tumours (H&E) and expression of α-SMA and p63 were analysed at the indicated time points after injection (n=5 animals). Scale bar= 100. B. Representative image of glucocorticoid receptor immunohistochemistry in in vivo samples. Red arrow indicates myoepithelial positive cells for GR and black arrow shows positivity in epithelial cells. µm.
