# Supplementary material for: Glucocorticoids promote transition of ductal carcinoma in situ to invasive ductal carcinoma by inducing myoepithelial cell apoptosis
Source: Breast Cancer Res. 2018 Jul 4;20:65. doi: 10.1186/s13058-018-0977-z (PMC6032539; doi:10.1186/s13058-018-0977-z)
Supplement: Supplementary file 6 — Figure S3. Corticosterone effects on mammary epithelial cells viability, apoptosis and functional abilities. a MCF10A epithelial, primary myoepithelial cells and MCF10DCIS cell viability evaluated by MTT assay after 48h of increasing doses of corticosterone treatment (0.125-1.5 µM). b MCF10A epithelial, primary myoepithelial cells and MCF10DCIS determination of cell apoptosis in its different stages (early, apoptosis, late and total) after treatment with corticosterone 0-1 µM by the Annexin V method and measured by flow cytometry. All experiments were carried out in triplicate. Statistical analysis was made using ANOVA followed by Dunn’s multiple test. c and d. Influence of corticosterone treatment on 3D growth of primary epithelial and myoepithelial cells and on MCF10DCIS for 14 days by immunofluorescence. Treatment with corticosterone 1 µM or vehicle (methanol) was carried out from day 5 after seeding until day 14. c Upper part. Immunodetection of K14 (myoepithelial cells), K19 (epithelial cells), with hoechst used as nuclei counterstaining. Scale bar=50 µm. c Bottom part. Quantification of morphometric analysis in control group and corticosterone-treated group of number of acini formed and related quantification of disrupted acini per total number of acini. d Upper part. Immunofluorescence of laminin (basement membrane), K14 (myoepithelial cells) and hoechst to counterstain nuclei. Scale bar=100 µm. d Bottom part. Quantification of laminin intensity after treatment with corticosterone 1 µM or vehicle by Image J software comparison test. e Immunofluorescence in MCF10DCIS 3D growth of laminin (basement membrane), Muc1 (epithelial cells) and hoechst to stain the nuclei. Arrows indicated rupture points of the acini showed. F. Morphometric quantification of disrupted acini and acinar fusion and intensity of laminin determined by integrated density parameter of Image J software. Scale bar=50µm. All experiments were carried out in triplicate. Statistical analysis was ma [file 13058_2018_977_MOESM6_ESM.pptx]

## Slide 1
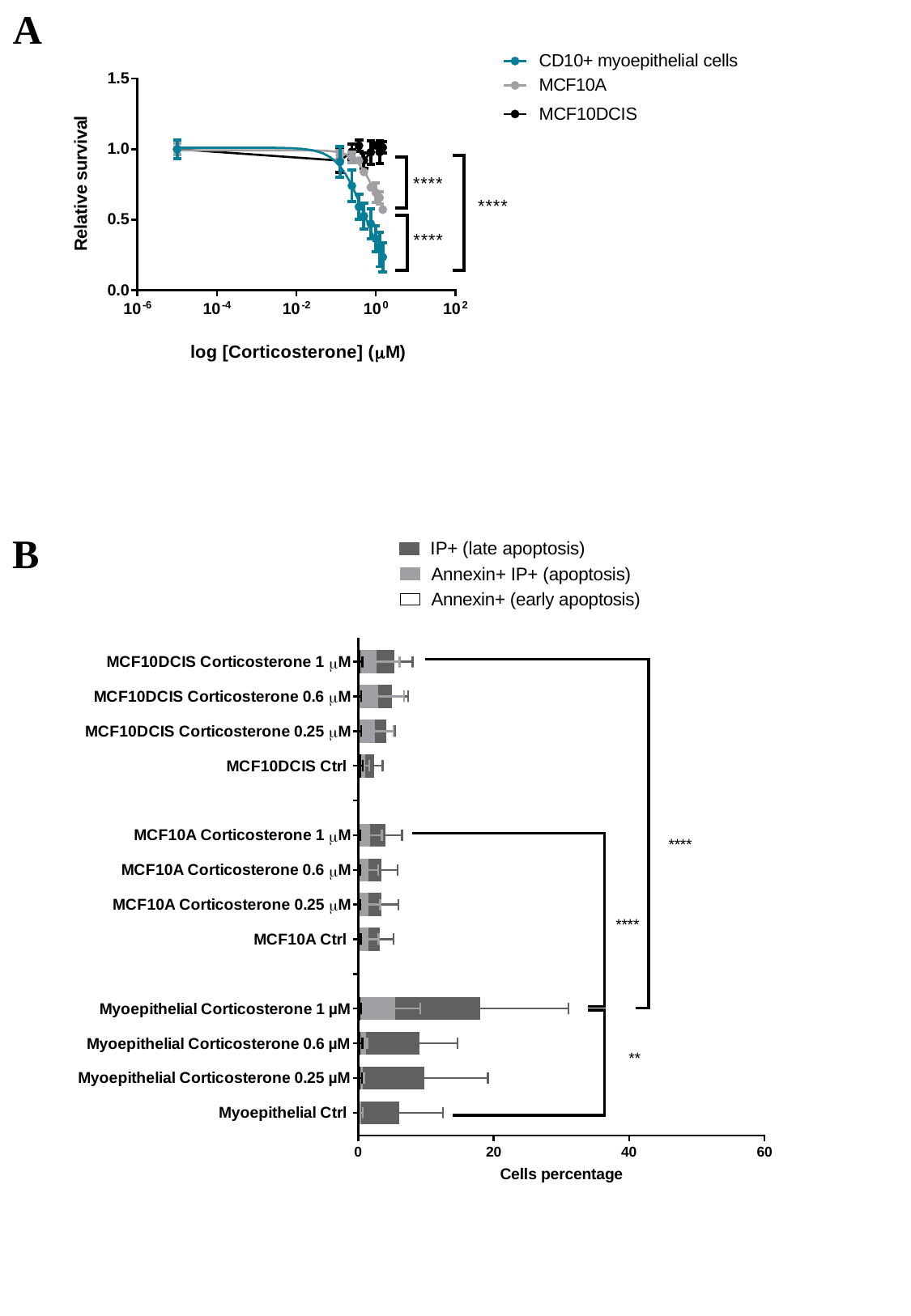

A
B

## Slide 2
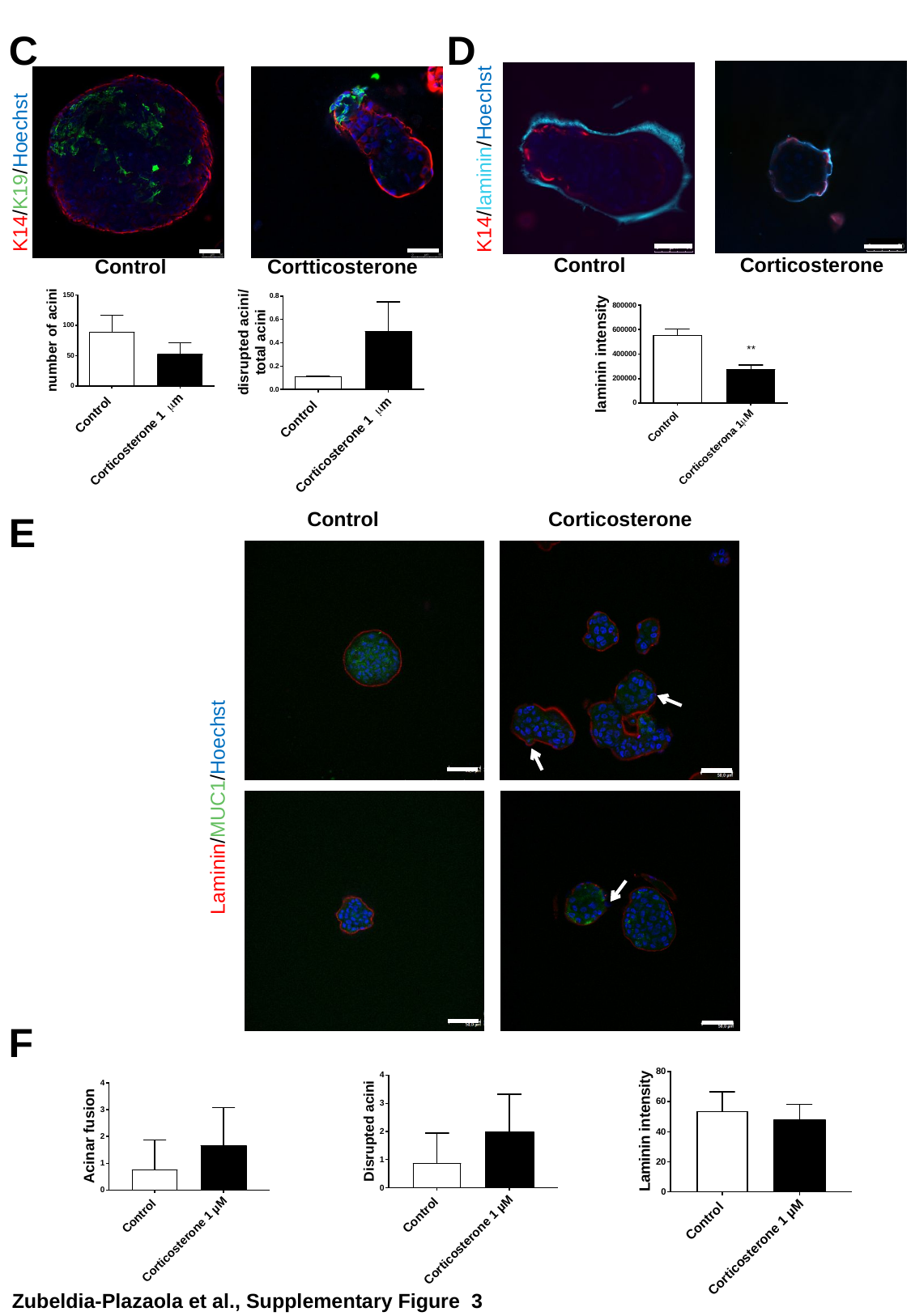

C
D
K14/laminin/Hoechst
K14/K19/Hoechst
Control
Corticosterone
Control
Cortticosterone
Control
Corticosterone
E
Laminin/MUC1/Hoechst
F
Zubeldia-Plazaola et al., Supplementary Figure 3

## Slide 3
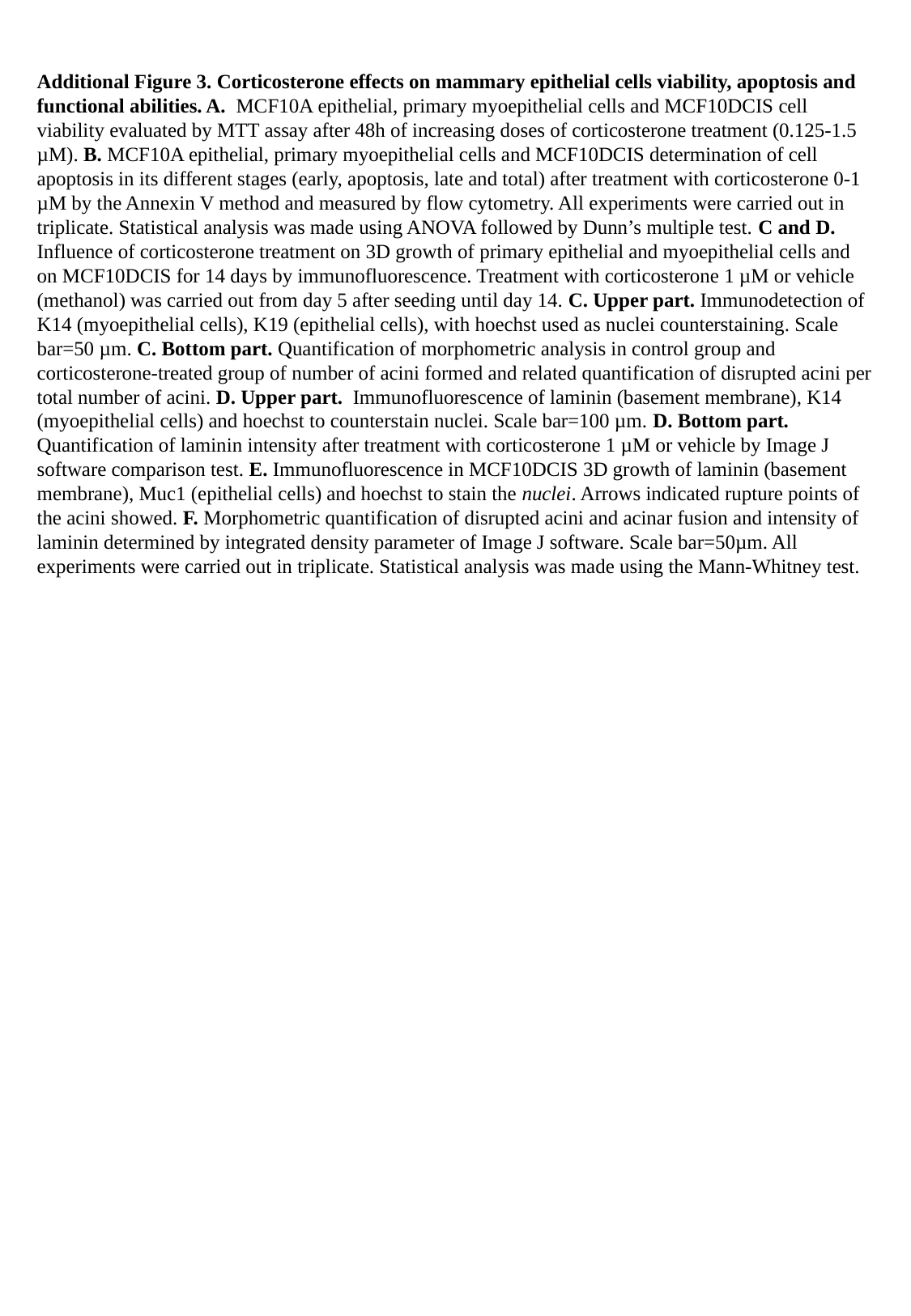

Additional Figure 3. Corticosterone effects on mammary epithelial cells viability, apoptosis and functional abilities. A. MCF10A epithelial, primary myoepithelial cells and MCF10DCIS cell viability evaluated by MTT assay after 48h of increasing doses of corticosterone treatment (0.125-1.5 µM). B. MCF10A epithelial, primary myoepithelial cells and MCF10DCIS determination of cell apoptosis in its different stages (early, apoptosis, late and total) after treatment with corticosterone 0-1 µM by the Annexin V method and measured by flow cytometry. All experiments were carried out in triplicate. Statistical analysis was made using ANOVA followed by Dunn’s multiple test. C and D. Influence of corticosterone treatment on 3D growth of primary epithelial and myoepithelial cells and on MCF10DCIS for 14 days by immunofluorescence. Treatment with corticosterone 1 µM or vehicle (methanol) was carried out from day 5 after seeding until day 14. C. Upper part. Immunodetection of K14 (myoepithelial cells), K19 (epithelial cells), with hoechst used as nuclei counterstaining. Scale bar=50 µm. C. Bottom part. Quantification of morphometric analysis in control group and corticosterone-treated group of number of acini formed and related quantification of disrupted acini per total number of acini. D. Upper part. Immunofluorescence of laminin (basement membrane), K14 (myoepithelial cells) and hoechst to counterstain nuclei. Scale bar=100 µm. D. Bottom part. Quantification of laminin intensity after treatment with corticosterone 1 µM or vehicle by Image J software comparison test. E. Immunofluorescence in MCF10DCIS 3D growth of laminin (basement membrane), Muc1 (epithelial cells) and hoechst to stain the nuclei. Arrows indicated rupture points of the acini showed. F. Morphometric quantification of disrupted acini and acinar fusion and intensity of laminin determined by integrated density parameter of Image J software. Scale bar=50µm. All experiments were carried out in triplicate. Statistical analysis was made using the Mann-Whitney test.
