# Supplementary material for: Glucocorticoids promote transition of ductal carcinoma in situ to invasive ductal carcinoma by inducing myoepithelial cell apoptosis
Source: Breast Cancer Res. 2018 Jul 4;20:65. doi: 10.1186/s13058-018-0977-z (PMC6032539; doi:10.1186/s13058-018-0977-z)
Supplement: Supplementary file 7 — Figure S4. Representative immunofluorescence images of MCF10DCIS xenografts in chicken embryo CAM membrane.a Hoechst in blue, p63 in red and laminin in green. b Merge of p63 and laminin double immunofluorescence images and zoom in showing an acini detail. Scale bar=20 µm. (PPT 4617 kb) [file 13058_2018_977_MOESM7_ESM.ppt]

## Slide 1
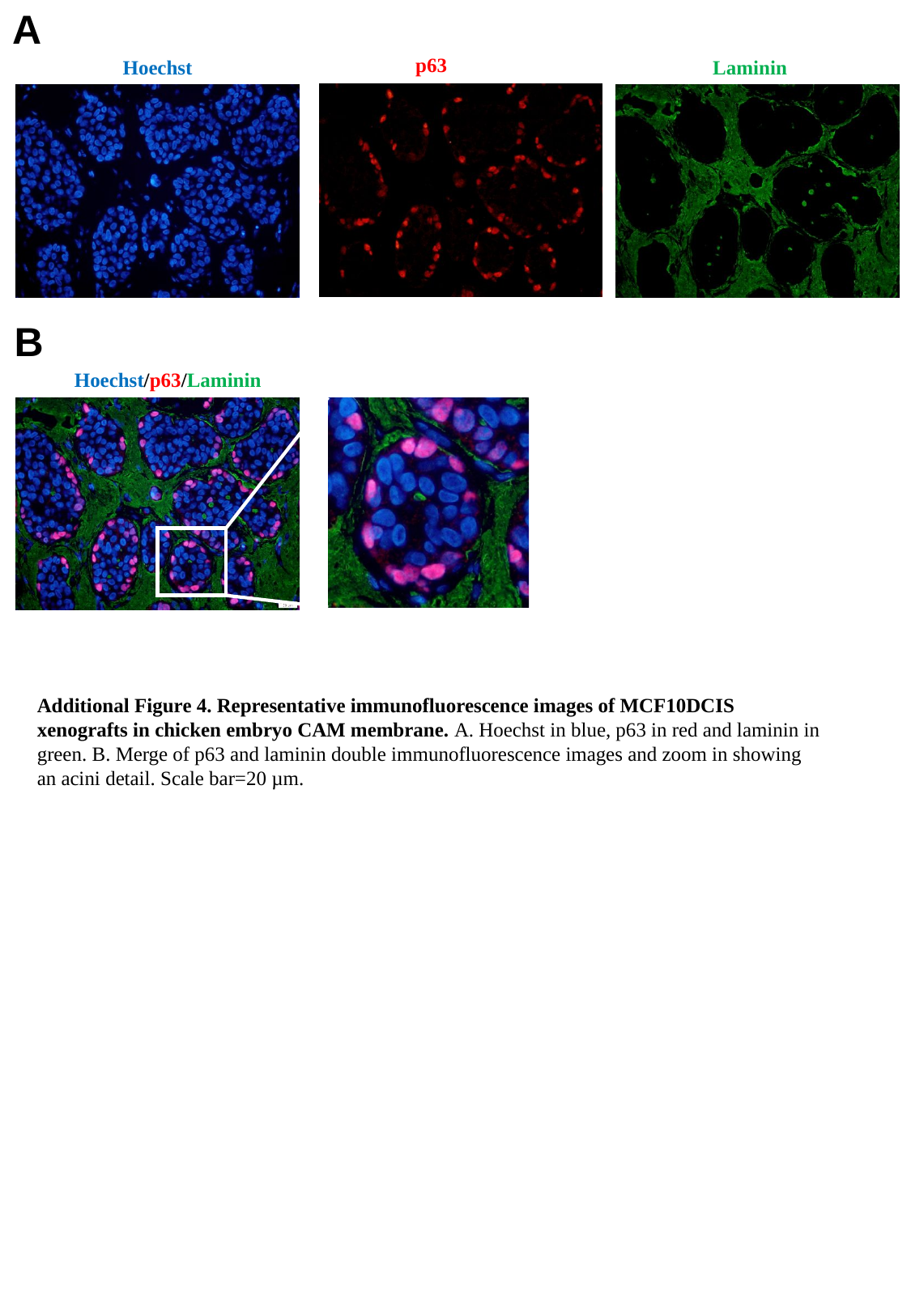

A
p63
Hoechst
Laminin
B
Hoechst/p63/Laminin
Additional Figure 4. Representative immunofluorescence images of MCF10DCIS xenografts in chicken embryo CAM membrane. A. Hoechst in blue, p63 in red and laminin in green. B. Merge of p63 and laminin double immunofluorescence images and zoom in showing an acini detail. Scale bar=20 µm.
