# Supplementary material for: Glucocorticoids promote transition of ductal carcinoma in situ to invasive ductal carcinoma by inducing myoepithelial cell apoptosis
Source: Breast Cancer Res. 2018 Jul 4;20:65. doi: 10.1186/s13058-018-0977-z (PMC6032539; doi:10.1186/s13058-018-0977-z)
Supplement: Supplementary file 8 — Figure S5. Representative immunofluorescence images of human samples of DCIS (6 patients) and DCIS + IDC (6 patients).a Double immunofluorescence of p63 (myoepithelial cells) and cleaved caspase 3 indicated with red arrows and hoechst as a nuclei counterstainer in DCIS sample patients and b in DCIS + IDC sample patients. White scale bar=20 and red scale bar=50 µm. (PPTX 13532 kb) [file 13058_2018_977_MOESM8_ESM.pptx]

## Slide 1
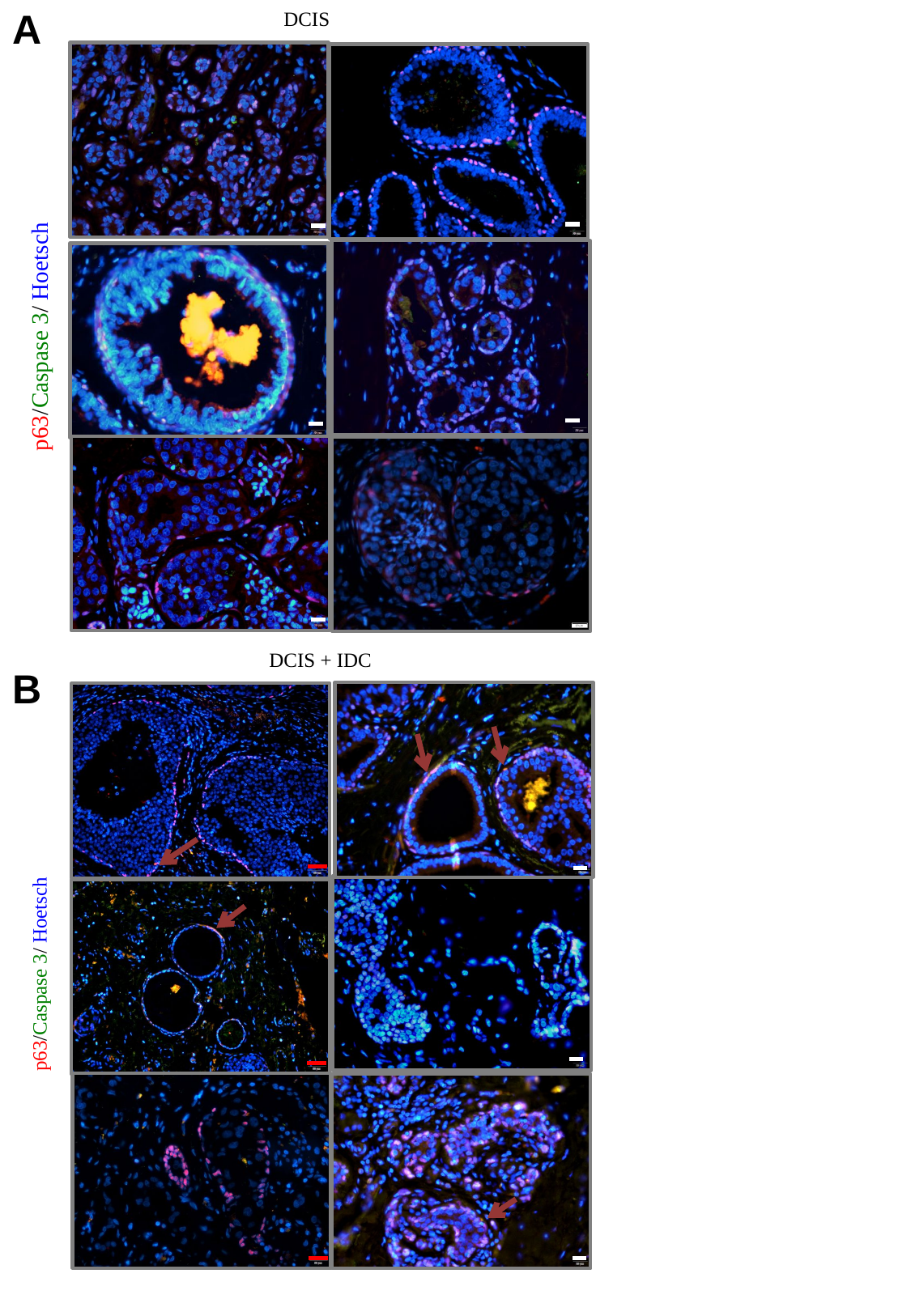

DCIS
A
p63/Caspase 3/ Hoetsch
DCIS + IDC
B
p63/Caspase 3/ Hoetsch

## Slide 2
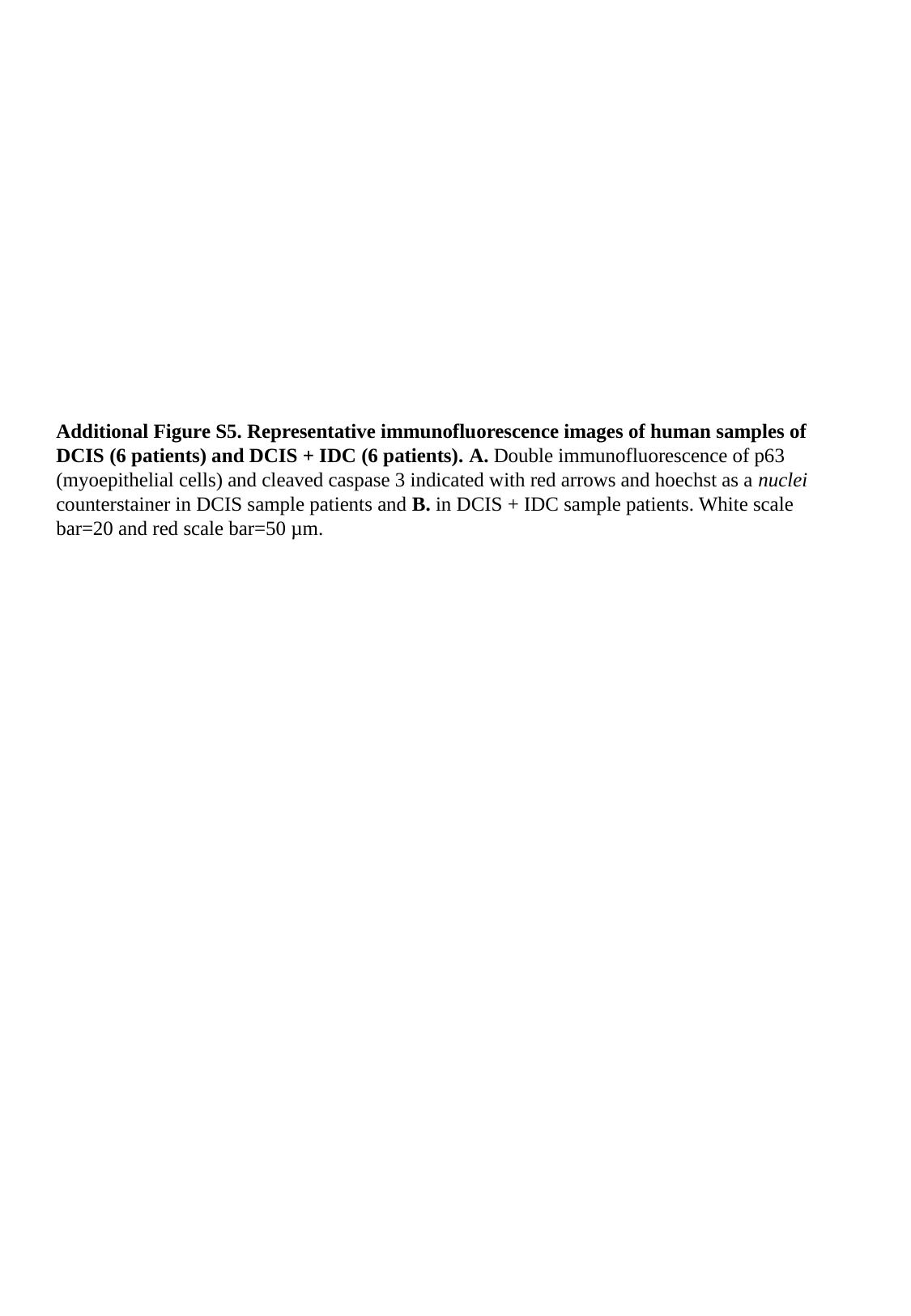

Additional Figure S5. Representative immunofluorescence images of human samples of DCIS (6 patients) and DCIS + IDC (6 patients). A. Double immunofluorescence of p63 (myoepithelial cells) and cleaved caspase 3 indicated with red arrows and hoechst as a nuclei counterstainer in DCIS sample patients and B. in DCIS + IDC sample patients. White scale bar=20 and red scale bar=50 µm.
